# An Improved Cubature Kalman Filter for GNSS-Denied and System-Noise-Varying INS/GNSS Navigation

**DOI:** 10.3390/mi16101116

**Published:** 2025-09-29

**Authors:** Di Liu, Xiyuan Chen, Bingbo Cui

**Affiliations:** 1 Key Laboratory of Micro-Inertial Instrument and Advanced Navigation Technology, Ministry of Education, School of Instrument Science and Engineering, Southeast University, Nanjing 210096, China; sdili_liudi@163.com; 2 School of Information and Automation, Qilu University of Technology (Shandong Academy of Sciences), Jinan 250353, China; 3 Key Laboratory of Modern Agricultural Equipment and Technology, Ministry of Education and Jiangsu Province, Jiangsu University, Zhenjiang 212013, China; cuibingbo@ujs.edu.cn

**Keywords:** integrated navigation, INS/GNSS, GNSS-denied, navigation system uncertainty

## Abstract

The degradation of nonlinear filtering in INS/GNSS integrated navigation due to missing GNSS observations and system noise uncertainty is addressed in this paper. An improved cubature Kalman filter (ICKF) is proposed, leveraging a modified cubature point update framework (MUF) and the maximum likelihood (ML) principle. In the ICKF, the ML principle is employed to estimate the process noise covariance, which is then integrated into the MUF to construct the posterior cubature points directly, bypassing the need for resampling. As the process noise covariance is updated in real time, and the prediction cubature points’ error is directly transferred to the posterior cubature points, the proposed algorithm demonstrates reduced sensitivity to missing observations and system noise uncertainty. The effectiveness of the proposed algorithm has been validated through both simulation and practical experiments.

## 1. Introduction

The inertial navigation system (INS) is an autonomous navigation system that provides the attitude, velocity, and position of a carrier using data from inertial sensors [1,2,3,4]. However, errors introduced by inertial sensors accumulate over time, which hampers the INS from meeting long-range and high-precision navigation requirements [5,6,7,8]. The Global Navigation Satellite System (GNSS) is a space-based radio navigation system that provides high-precision navigation information without cumulative errors over time [9,10,11]. However, GNSS suffers from a low update frequency and is vulnerable to environmental interference [12,13,14]. Due to their complementary characteristics, the integration of INS and GNSS is widely employed for high-performance navigation [15,16,17].

The accuracy of INS/GNSS navigation primarily depends on two factors: continuous observation data and prior statistical information on process noise [18,19,20]. In complex environments, missing observations frequently occur in GNSS, leading to the degradation of INS/GNSS integrated navigation performance [21,22,23].

Consequently, numerous methods have been proposed to enhance INS/GNSS navigation performance during GNSS outages, including data-driven methods (DDMs) and machine learning methods (MLMs) [24]. In DDMs, data-driven schemes support various extensions of Kalman filters (KFs) to bridge signal outages. However, this approach suffers from overfitting, challenging parameter settings, and poor real-time performance, and its performance is inferior to that of KF during short outages [12,25]. To overcome the shortcomings of DDMs, MLMs have been proposed to improve navigation performance. MLMs have been extensively used in fuzzy control, state estimation, and data sampling for stability and adaptive synchronization applications [26]. It is well established that the classical three-layer fully connected neural network (NN) can approximate any nonlinear feature. Based on this theory, MLMs are employed to establish the relationship between INS parameters and GNSS information and can subsequently predict relevant parameters as GNSS observations during outages [22]. MLMs include the Grey predictor [24,27], radial basis function NN [28,29], long short-term memory NN [12,22], wavelet NN [30], and multilayer perceptron NN [31]. Generally, these methods still struggle to provide accurate navigation information during prolonged GNSS outages [12].

The prior statistical information on process noise is another significant factor influencing the quality of INS/GNSS navigation, describing the uncertainty associated with the system process model [32]. Since process models are only theoretical approximations of actual systems, errors are inevitable. Moreover, because of the coupling relationship between process noise and the system model, ensuring the accuracy of the prior statistical information on process noise is challenging. This coupling arises because the process noise term, ${\bar{w}}_{k-1}$ in Equation (1), must account for the aggregate of all unmodeled dynamics, linearization errors, and external disturbances not captured by the nominal system function f(⋅). Therefore, any inaccuracy in f(⋅) directly contaminates the characterization of ${\bar{w}}_{k-1}$. For instance, an overly simplified dynamic model would necessitate a larger process noise covariance ${\bar{Q}}_{k-1}$ to compensate for the missing dynamics, while a highly accurate model would require a smaller ${\bar{Q}}_{k-1}$. However, in practice, the true system dynamics are unknown and time varying (e.g., due to changing vehicle maneuvers or environmental conditions), making it difficult to pre-determine a fixed and accurate ${\bar{Q}}_{k-1}$ matrix. This interdependence between model fidelity and noise statistics is the essence of the coupling, which complicates the acquisition of reliable prior noise information. Consequently, adaptive filters have been proposed to enhance the robustness of navigation systems against process model uncertainty, including the strong tracking filter [16,33], ML-based filter [7,32], Sage–Husa adaptive Kalman filter [34], and variational Bayesian Kalman filter [20,32].

The strong tracking filter adaptively adjusts the filter gain matrix by introducing an adaptive fading factor, ensuring that the residual sequence remains orthogonal. Consequently, performance degradation due to model uncertainty can be effectively mitigated. However, the strong tracking filter does not account for the impact of measurement abnormalities, leading to severe degradation in filtering performance when such abnormalities occur. The ML-based filter is unbiased in achieving minimum covariance when measurements are independent and identically distributed. Nonetheless, existing ML-based filtering methods are developed specifically for linear systems, rendering them unsuitable for nonlinear state estimation. The classical Sage–Husa adaptive Kalman filter is based on the principle of covariance matching [35]. However, it does not guarantee that noise statistics will converge to the correct covariance matrices. The variational Bayesian Kalman filter primarily estimates the variance of measurement noise and achieves better performance in linear state space models, whereas research on process model uncertainty and nonlinear state space models is limited.

Besides the aforementioned filters, another line of research employs robust estimation theory, such as Huber’s M-estimation, to enhance filter resilience against observation outliers and missing measurements. For instance, Sahl et al. [36] developed a robust CKF for moving-target tracking in the presence of missing measurements by integrating M-estimation methodology into the standard CKF framework. However, this approach primarily addresses uncertainties originating from the measurement model and does not tackle the critical issue of time-varying process noise uncertainty, which is paramount in INS/GNSS applications.

In INS/GNSS integrated navigation applications, it is common for missing observations and process model uncertainty to coexist. To the authors’ knowledge, there are few studies on this problem. Cui et al. [37] proposed a resampling-free sigma-point update framework (RSUF) based on process model uncertainty analysis, which is embedded into the spherical–radial rule to update the posterior sigma points, thereby enhancing the robustness of the cubature Kalman filter (CKF) against missing observations and process model uncertainty. However, when process model uncertainty is significant, this method separates the system’s noise variance from the sigma point update process, leading to a less effective sigma point error matrix.

Motivated by the fact that the ML principle has proven effective in estimating unknown parameters of statistical models, we study the combination of ML estimation and RSUF in this paper. The ML principle is utilized to enhance the robustness of the CKF based on RSUF, which not only improves the robustness of the measurement update to observation loss but also reduces the sensitivity of RSUF to process model uncertainty.

The primary contributions of this paper are as follows: (1) Due to the challenges of theoretical derivation, there has been limited exploration of applying ML estimation to enhance the performance of CKF in nonlinear state estimation, particularly in the presence of process model uncertainty. We propose a novel algorithm that extends ML estimation from linear Kalman filters to nonlinear CKF to adapt the process noise covariance. (2) We incorporate adaptive estimation into the resampling-free CKF framework to develop an improved CKF, which is further analyzed theoretically in terms of stability and computational complexity. (3) The proposed algorithm addresses the issue of nonlinear filtering degradation for INS/GNSS integrated navigation in the presence of both GNSS observation loss and process model uncertainty.

This paper is structured as follows: In Section 2, the problem of CKF is formulated. In Section 3, the ICKF algorithm is derived based on the ML principle and MUF, and the computational complexity and stability of ICKF are analyzed. In Section 4, the results of the simulation and field test are presented. Finally, the conclusions of the paper are drawn in Section 5.

## 2. Problem Formulation

Consider the nonlinear discrete-time system model of an INS/GNSS, which is described as follows [37]:

(1) $${\bar{x}}_{k}=f({\bar{x}}_{k-1})+{\bar{w}}_{k-1}$$

(2) $${\bar{z}}_{k}=h({\bar{x}}_{k})+{\bar{v}}_{k}$$

where ${\bar{x}}_{k}\in R^{\bar{n}}$ and ${\bar{z}}_{k}\in R^{\bar{m}}$ denote the $\bar{n}$-dimensional state and the $\bar{m}$-dimensional measurement at instant k, respectively. ${\bar{w}}_{k-1}$ and ${\bar{v}}_{k}$ represent the process noise and measurement noise with ${\bar{w}}_{k-1}∼(0,{\bar{Q}}_{k-1})$ and ${\bar{v}}_{k}∼(0,{\bar{R}}_{k})$. ${\bar{Q}}_{k-1}$ and ${\bar{R}}_{k}$ denote the covariance of ${\bar{w}}_{k-1}$ and ${\bar{v}}_{k}$, respectively. f(⋅) denotes the dynamic function and h(⋅) denotes the measurement function. In the following derivations, we employ a notation convention where a hat symbol (︹) denotes estimated quantities (e.g., ${\overset{︹}{x}}_{k\left|k-1\right.}$ for the predicted state mean) and a bar symbol (¯) denotes predicted or propagated quantities (e.g., ${\bar{P}}_{k\left|k-1\right.}$ for the predicted covariance, ${\bar{X}}_{i}$ for propagated cubature points). The specific definitions of each symbolized variable are provided upon their first appearance. Given the posterior probability density function (PDF) $p({\bar{x}}_{k-1})=N({\bar{x}}_{k-1};{\overset{︹}{x}}_{k-1\left|k-1\right.},{\bar{P}}_{k-1\left|k-1\right.})$ at instant k−1 and the observation ${\bar{z}}_{k}$ at instant k, by calculating the first and second moments of the Gaussian distribution of the posterior PDF $p({\bar{x}}_{k})$ at instant k, $p({\bar{x}}_{k})$ is approximated as $p({\bar{x}}_{k})=N({\bar{x}}_{k};{\overset{︹}{x}}_{k\left|k\right.},{\bar{P}}_{k\left|k\right.})$, with the moments involved in the approximation process represented as follows.

To approximate the first two moments (mean and covariance) of the predicted state, which are intractable due to the nonlinearity, the following Gaussian integrals must be computed:

(3) $${\overset{︹}{x}}_{k\left|k-1\right.}=\int f({\bar{x}}_{k-1})N({\bar{x}}_{k-1};{\overset{︹}{x}}_{k-1\left|k-1\right.},{\bar{P}}_{k-1\left|k-1\right.})d{\bar{x}}_{k-1}$$

Equation (3) defines the predicted state mean ${\overset{︹}{x}}_{k\left|k-1\right.}$ by calculating its expectation. It is crucial to note that this integral formulation does not imply that the physical motion of the object is governed by the probability density function (PDF). Instead, the object’s true kinematics are described by the nonlinear system function f(⋅) in Equation (1). However, since the previous state estimate ${\overset{︹}{x}}_{k-1\left|k-1\right.}$ is not a deterministic value but is characterized by a distribution (the posterior PDF $N({\bar{x}}_{k-1};{\overset{︹}{x}}_{k-1\left|k-1\right.},{\bar{P}}_{k-1\left|k-1\right.})$), the optimal predictor for the next state is the expectation of $f({\bar{x}}_{k-1})$ over this distribution. In essence, Equation (3) propagates the uncertainty of the state through the system dynamics to obtain the best possible prediction in a Bayesian mean-squared error sense.

(4) $${\bar{P}}_{k\left|k-1\right.}=\int \left\{f({\bar{x}}_{k-1})-{\overset{︹}{x}}_{k\left|k-1\right.}\right\}{\left\{f({\bar{x}}_{k-1})-{\overset{︹}{x}}_{k\left|k-1\right.}\right\}}^{T}N({\bar{x}}_{k-1};{\overset{︹}{x}}_{k-1\left|k-1\right.},{\bar{P}}_{k-1\left|k-1\right.})d{\bar{x}}_{k-1}+{\bar{Q}}_{k-1}$$

Similarly, the moments of the measurement prediction can be formulated as:

(5) $${\overset{︹}{z}}_{k\left|k-1\right.}=\int h({\bar{x}}_{k})N({\bar{x}}_{k};{\overset{︹}{x}}_{k\left|k-1\right.},{\bar{P}}_{k\left|k-1\right.})d{\bar{x}}_{k}$$

Note that this integral represents the expectation of the measurement function over the predicted state distribution, which is fundamental to nonlinear Bayesian filtering. It does not imply that the physical measurement process involves integration.

(6) $${\bar{P}}_{\bar{z}\bar{z},k\left|k-1\right.}=\int \left\{h({\bar{x}}_{k})-{\overset{︹}{z}}_{k\left|k-1\right.}\right\}{\left\{h({\bar{x}}_{k})-{\overset{︹}{z}}_{k\left|k-1\right.}\right\}}^{T}N({\bar{x}}_{k};{\overset{︹}{x}}_{k\left|k-1\right.},{\bar{P}}_{k\left|k-1\right.})d{\bar{x}}_{k}+{\bar{R}}_{k}$$

(7) $${\bar{P}}_{\bar{x}\bar{z},k\left|k-1\right.}=\int \left\{{\bar{x}}_{k}-{\overset{︹}{x}}_{k\left|k-1\right.}\right\}{\left\{h({\bar{x}}_{k})-{\overset{︹}{z}}_{k\left|k-1\right.}\right\}}^{T}N({\bar{x}}_{k};{\overset{︹}{x}}_{k\left|k-1\right.},{\bar{P}}_{k\left|k-1\right.})d{\bar{x}}_{k}$$

where ${\bar{P}}_{k\left|k-1\right.}$ is the predicted state error covariance matrix, representing the uncertainty of the state prediction. ${\bar{P}}_{\bar{z}\bar{z},k\left|k-1\right.}$ is the innovation covariance matrix, representing the uncertainty of the measurement prediction. ${\bar{P}}_{\bar{x}\bar{z},k\left|k-1\right.}$ is the cross-covariance matrix between the state and measurement predictions. ${\bar{P}}_{k\left|k\right.}$ is the updated state error covariance matrix, representing the uncertainty of the state estimate after incorporating the measurement.

Note that the above process involves the calculation of integrals. The CKF was proposed based on the third-degree spherical–radial cubature rule to approximate the integral. The filtering process of the CKF includes a time update and a measurement update, as follows.

(1) Time Update

Given the state estimate ${\overset{︹}{x}}_{k-1\left|k-1\right.}$ and its corresponding error covariance ${\bar{P}}_{k-1\left|k-1\right.}$ at instant k−1. According to Equations (8) and (9), the cubature points are generated.

(8) $${\bar{S}}_{k-1|k-1}=chol({\bar{P}}_{k-1|k-1})$$

(9) $${\bar{X}}_{i,k-1\left|k-1\right.}={\bar{S}}_{k-1\left|k-1\right.}{\bar{\xi }}_{i}+{\overset{︹}{x}}_{k-1\left|k-1\right.},i=1,\ldots ,2\bar{n}$$

where chol(⋅) represents the Cholesky decomposition, ${\bar{\xi }}_{i}=\left\{\begin{array}{l} \sqrt{\bar{n}}e_{i},i=1,\ldots ,\bar{n}\\ -\sqrt{\bar{n}}e_{i},i=\bar{n}+1,\ldots ,2\bar{n} \end{array}\right.$, and $e_{i}$ denotes the i-th column of the $\bar{n}\times \bar{n}$ identity matrix.

The propagated cubature points are generated through the application of the state function.

(10) $${\bar{X}}_{i,k\left|k-1\right.}^{*}=f({\bar{X}}_{i,k-1\left|k-1\right.})$$

The predicted state and covariance are computed as follows:

(11) $${\overset{︹}{x}}_{k\left|k-1\right.}=\sum_{i=1}^{2\bar{n}}{\bar{\omega }}_{i}({\bar{X}}_{i,k\left|k-1\right.}^{*})$$

(12) $${\bar{P}}_{k\left|k-1\right.}=\sum_{i=1}^{2\bar{n}}{\bar{\omega }}_{i}({\bar{X}}_{i,k\left|k-1\right.}^{*}-{\overset{︹}{x}}_{k\left|k-1\right.}){\left({\bar{X}}_{i,k\left|k-1\right.}^{*}-{\overset{︹}{x}}_{k\left|k-1\right.}\right)}^{T}+{\bar{Q}}_{k-1}$$

where ${\bar{\omega }}_{i}$ denotes the weight of the cubature point and ${\bar{\omega }}_{i}=1/2\bar{n}$.

(2) Measurement Update

The updated cubature point based on the predicted state and covariance matrix is expressed as follows:

(13) $${\bar{S}}_{k|k-1}=chol({\bar{P}}_{k|k-1})$$

(14) $${\bar{X}}_{i,k\left|k-1\right.}={\bar{S}}_{k\left|k-1\right.}{\bar{\xi }}_{i}+{\overset{︹}{x}}_{k\left|k-1\right.}$$

The cubature point propagated via the measurement function is expressed as follows:

(15) $${\bar{Z}}_{i,k\left|k-1\right.}=h({\bar{X}}_{i,k\left|k-1\right.})$$

Based on the propagated cubature point, the predicted measurement, covariance, and cross-covariance are obtained.

(16) $${\overset{︹}{z}}_{k\left|k-1\right.}=\sum_{i=1}^{2\bar{n}}{\bar{\omega }}_{i}{\bar{Z}}_{i,k\left|k-1\right.}$$

(17) $${\bar{P}}_{\bar{z}\bar{z},k\left|k-1\right.}=\sum_{i=1}^{2\bar{n}}{\bar{\omega }}_{i}({\bar{Z}}_{i,k\left|k-1\right.}-{\overset{︹}{z}}_{k\left|k-1\right.}){\left({\bar{Z}}_{i,k\left|k-1\right.}-{\overset{︹}{z}}_{k\left|k-1\right.}\right)}^{T}+{\bar{R}}_{k}$$

(18) $${\bar{P}}_{\bar{x}\bar{z},k\left|k-1\right.}=\sum_{i=1}^{2\bar{n}}{\bar{\omega }}_{i}({\bar{X}}_{i,k\left|k-1\right.}^{*}-{\overset{︹}{x}}_{k\left|k-1\right.}){\left({\bar{Z}}_{i,k\left|k-1\right.}-{\overset{︹}{z}}_{k\left|k-1\right.}\right)}^{T}$$

The Kalman gain is formulated as follows:

(19) $${\bar{K}}_{k}={\bar{P}}_{\bar{x}\bar{z},k\left|k-1\right.}{\bar{P}}_{\bar{z}\bar{z},k\left|k-1\right.}^{-1}$$

The state and error covariance matrix are updated via Equations (20) and (21).

(20) $${\overset{︹}{x}}_{k\left|k\right.}={\overset{︹}{x}}_{k\left|k-1\right.}+{\bar{K}}_{k}({\bar{z}}_{k}-{\overset{︹}{z}}_{k\left|k-1\right.})$$

(21) $${\bar{P}}_{k\left|k\right.}={\bar{P}}_{k\left|k-1\right.}-{\bar{K}}_{k}{\bar{P}}_{\bar{z}\bar{z},k\left|k-1\right.}{\bar{K}}_{k}^{T}$$

**Remark** **1.** *In the implementation of CKF, the information transferred from the time update process to the measurement update process is the mean and variance of the prior PDF, and the state posterior PDF of the measurement update output is used as the input of the time update process to start a new filtering period. When the observations are missing and the process model has uncertainty, the state posterior PDF entering the next time update process has a large error, and the cubature point produced by the PDF to approximate the state prior PDF of the system will produce a larger error, which then affects the accuracy of calculating the Gaussian integrals involved in the prediction and update steps.*

**Remark** **2.** *When uncertainty exists within the process model, the covariance of the system noise ceases to be a fixed value. Additionally, when an observation is missing, the CKF operates in prediction mode, and $\left\{\begin{array}{l} p({\bar{x}}_{k}\left|{\overset{︹}{Z}}_{k-1}\right.)=\int p({\bar{x}}_{k}\left|{\bar{x}}_{k-1}\right.)p({\bar{x}}_{k-1}\left|{\bar{z}}_{k-1}\right.)d{\bar{x}}_{k-1}\\ {\overset{︹}{Z}}_{k-1}=\{{\bar{z}}_{i},i=1,\ldots ,k-1\} \end{array}\right.$ becomes the sole state constraint equation during the estimation process. Determining how to fully utilize the information generated by this equation is crucial for enhancing the robustness of the CKF.*

The above formulation reveals two inherent limitations of the standard CKF in practical INS/GNSS applications. First, as noted in Remark 1, the filter’s performance degrades significantly during GNSS outages because the erroneous posterior PDF from the previous cycle is directly used to generate cubature points for the next prediction, leading to rapidly accumulating errors. Second, as indicated in Remark 2, the CKF assumes a fixed and known process noise covariance $\bar{Q}$. However, this assumption is often violated in real-world systems due to model simplifications and time-varying uncertainties. These two challenges—missing observations and process noise uncertainty—often occur simultaneously but are not adequately addressed by the standard CKF. This motivates the development of an improved algorithm that can jointly tackle these issues, which is presented in the following section.

## 3. Improved Cubature Kalman Filter Based on MUF and ML Principle

### 3.1. Design of the Proposed Algorithm

This section proposes an improved CKF based on the MUF and ML principle to mitigate the impact of process model uncertainty and missing observations on filtering performance. The proposed algorithm uses the ML principle to update the process noise covariance in real time and the MUF to directly transform the cubature point error matrix of the prior PDF to obtain the state posterior cubature point error matrix and regenerate cubature points. The detailed derivation process of the algorithm is outlined as follows.

The conditional PDF of the measurement is constructed using statistical data from the innovation, after which a fixed-length memory estimation window is applied within the ML principle to estimate the process noise covariance. First, the conditional PDF of the measurement is expressed as

(22) $$p{\left(\bar{z}\left|\bar{Q}\right.\right)}_{k}=\frac{1}{\sqrt{{\left(2\pi \right)}^{\bar{m}}\left|{\bar{P}}_{\bar{z}\bar{z},k\left|k-1\right.}\right|}}\exp(-\frac{1}{2}{\tilde{\tilde{z}}}_{k}^{T}{\bar{P}}_{\bar{z}\bar{z},k\left|k-1\right.}^{-1}{\tilde{\tilde{z}}}_{k})$$

where ${\tilde{\tilde{z}}}_{k}$ represents the innovation at instant k and ${\tilde{\tilde{z}}}_{k}={\bar{z}}_{k}-{\overset{︹}{z}}_{k\left|k-1\right.}$.

The likelihood function $L(\bar{Q}\left|{\bar{z}}_{k-\bar{N}+1:k}\right.)$ for $\left({\bar{z}}_{k-\bar{N}+1},{\bar{z}}_{k-\bar{N}+2},\cdots ,{\bar{z}}_{k}\right)$ is calculated by

(23) $$L(\bar{Q}\left|{\bar{z}}_{k-\bar{N}+1:k}\right.)=\prod_{j=k-\bar{N}+1}^{k}p(\bar{z}\left|\bar{Q}{)}_{j}\right.=\prod_{j=k-\bar{N}+1}^{k}\frac{1}{\sqrt{{\left(2\pi \right)}^{\bar{m}}\left|{\bar{P}}_{\bar{z}\bar{z},j\left|j-1\right.}\right|}}\exp(-\frac{1}{2}{\tilde{\tilde{z}}}_{j}^{T}{\bar{P}}_{\bar{z}\bar{z},j\left|j-1\right.}^{-1}{\tilde{\tilde{z}}}_{j})$$

where $\bar{N}$ represents the size of the fixed-length memory estimation window.

**Remark** **3.** *The derivation of the ML estimator assumes a constant dimension* $\bar{m}$ *of the innovation vector* ${\tilde{\tilde{z}}}_{j}$ *across the estimation window* $\bar{N}$*. In the context of this work (tightly coupled INS/GNSS), this is typically maintained by the navigation system through measurement selection and validation routines, even if the number of available satellites fluctuates. For systems involving heterogeneous sensors with fundamentally different and varying measurement dimensions (e.g., fusing GNSS with a magnetometer), the formulation would require adaptation, such as employing a composite likelihood approach. This is considered beyond the scope of this paper but represents a valuable direction for future work.*

According to the ML principle, the estimation problem of covariance $\bar{Q}$ can be transformed into solving the following optimization problem:

(24) $${\bar{Q}}_{ML}=\arg\underset{\bar{Q}}{\max}\left\{L(\bar{Q}\left|{\bar{z}}_{k-\bar{N}+1:k}\right.)\right\}$$

By applying logarithmic transformations to both sides of Equation (24) and omitting the constant term, it can be further rewritten as Equation (25).

(25) $${\bar{Q}}_{ML}=\arg\underset{\bar{Q}}{\min}\left[\sum_{j=k-\bar{N}+1}^{k}\ln(\left|{\bar{P}}_{\bar{z}\bar{z},j\left|j-1\right.}\right|)+\sum_{j=k-\bar{N}+1}^{k}{\tilde{\tilde{z}}}_{j}^{T}{\bar{P}}_{\bar{z}\bar{z},j\left|j-1\right.}^{-1}{\tilde{\tilde{z}}}_{j}\right]$$

To facilitate solving the optimization problem shown in Equation (25), we define the following cost function.

(26) $$J(\bar{Q})=\sum_{j=k-\bar{N}+1}^{k}\left\{\ln(\left|{\bar{P}}_{\bar{z}\bar{z},j\left|j-1\right.}\right|)+{\tilde{\tilde{z}}}_{j}^{T}{\bar{P}}_{\bar{z}\bar{z},j\left|j-1\right.}^{-1}{\tilde{\tilde{z}}}_{j}\right\}$$

Taking the partial derivatives of the above cost function with respect to each element of $\bar{Q}$ and setting them equal to 0, the following maximum likelihood equation can be obtained:

(27) $$\sum_{j=k-\bar{N}+1}^{k}\left\{\begin{array}{l} tr\left[{\bar{P}}_{\bar{z}\bar{z},j\left|j-1\right.}^{-1}\frac{\partial {\bar{P}}_{\bar{z}\bar{z},j\left|j-1\right.}}{\partial {\bar{Q}}_{il}}\right]-\\ {\tilde{\tilde{z}}}_{j}^{T}{\bar{P}}_{\bar{z}\bar{z},j\left|j-1\right.}^{-1}\frac{\partial {\bar{P}}_{\bar{z}\bar{z},j\left|j-1\right.}}{\partial {\bar{Q}}_{il}}{\bar{P}}_{\bar{z}\bar{z},j\left|j-1\right.}^{-1}{\tilde{\tilde{z}}}_{j} \end{array}\right\}=0$$

where $i,l=1,2,\cdots ,\bar{n}$, tr(⋅) denotes the trace operation. ${\bar{Q}}_{il}$ represents the i-th row and l-th column element in $\bar{Q}$.

**Remark** **4.** *Although Equation (25) involves the measurement innovation and its covariance, the optimization variable is the process noise covariance matrix* $\bar{Q}$*, which is of size* $\bar{n}\times \bar{n}$*. The resulting estimate* ${\bar{Q}}_{ML}$ *in Equation (42) is therefore also an* $\bar{n}\times \bar{n}$ *matrix, ensuring dimensional consistency.*

By applying a Taylor expansion to the state estimation ${\overset{︹}{x}}_{k-1\left|k-1\right.}$ at instant k−1, the prediction error of the state at instant k is expressed as follows:

(28) $${\tilde{\tilde{x}}}_{k\left|k-1\right.}={\bar{F}}_{k}{\tilde{\tilde{x}}}_{k-1\left|k-1\right.}+\Delta {\tilde{\tilde{x}}}_{k-1\left|k-1\right.}+{\bar{w}}_{k}$$

where ${\bar{F}}_{k}=\frac{\partial f(\bar{x})}{\partial \bar{x}}{|}_{\bar{x}={\overset{︹}{x}}_{k-1\left|k-1\right.}}$, $\Delta {\tilde{\tilde{x}}}_{k-1\left|k-1\right.}$ represents the second- and higher-order terms in the Taylor expansion coefficients and ${\tilde{\tilde{x}}}_{k-1\left|k-1\right.}$ denotes the estimation error of the state at instant k−1.

To simplify the error expression, an unknown time-varying matrix ${\bar{\beta }}_{k}=diag({\bar{\beta }}_{1,k},{\bar{\beta }}_{2,k},\cdots ,{\bar{\beta }}_{\bar{n},k})$ is introduced to express the first-order linear error in Equation (28); therefore, Equation (28) can be rewritten as

(29) $${\tilde{\tilde{x}}}_{k\left|k-1\right.}={\bar{\beta }}_{k}{\bar{F}}_{k}{\tilde{\tilde{x}}}_{k-1\left|k-1\right.}+{\bar{w}}_{k}$$

where diag(⋅) denotes the diagonal operation of the matrix.

Furthermore, the state prediction covariance can be expressed as

(30) $$\begin{array}{l} {\bar{P}}_{k\left|k-1\right.}=E\left[{\tilde{\tilde{x}}}_{k\left|k-1\right.}{\tilde{\tilde{x}}}_{k\left|k-1\right.}^{T}\right]\\ \;=E\left[({\bar{\beta }}_{k}{\bar{F}}_{k}{\tilde{\tilde{x}}}_{k-1\left|k-1\right.}+{\bar{w}}_{k}){\left({\bar{\beta }}_{k}{\bar{F}}_{k}{\tilde{\tilde{x}}}_{k-1\left|k-1\right.}+{\bar{w}}_{k}\right)}^{T}\right]\\ \;={\bar{\beta }}_{k}{\bar{F}}_{k}{\bar{P}}_{k-1\left|k-1\right.}{\bar{F}}_{k}^{T}{\bar{\beta }}_{k}+\bar{Q} \end{array}$$

For the purpose of deriving the ML estimate of the process noise covariance, we temporarily assume a linear measurement model in this subsection. Note that the actual filtering still uses the nonlinear measurement function h(⋅).

(31) $${\bar{z}}_{k}={\bar{H}}_{k}{\bar{x}}_{k}+{\bar{v}}_{k}$$

where ${\bar{H}}_{k}$ represents the measurement matrix, ${\bar{z}}_{k}$ denotes measurement at instant k, ${\bar{v}}_{k}$ represents the measurement noise with ${\bar{v}}_{k}∼(0,{\bar{R}}_{k})$, and ${\bar{R}}_{k}$ denotes the covariance of ${\bar{v}}_{k}$.

Further, we can have

(32) $${\bar{P}}_{\bar{z}\bar{z},k\left|k-1\right.}={\bar{H}}_{k}{\bar{P}}_{k\left|k-1\right.}{\bar{H}}_{k}^{T}+{\bar{R}}_{k}$$

Solve the partial derivative for ${\bar{Q}}_{il}$ with Equations (30) and (31). When the filtering process in the estimation window tends to stabilize, the first-order term of $\frac{\partial {\bar{P}}_{k\left|k-1\right.}}{\partial {\bar{Q}}_{il}}$ (where i and l are the row and column indices, respectively, of the process noise covariance matrix $\bar{Q}$) can be ignored, and the following equation is obtained:

(33) $$\frac{\partial {\bar{P}}_{\bar{z}\bar{z},k\left|k-1\right.}}{\partial {\bar{Q}}_{il}}={\bar{H}}_{k}\frac{\partial \bar{Q}}{\partial {\bar{Q}}_{il}}{\bar{H}}_{k}^{T}$$

Substituting Equation (33) into Equation (27), we obtain

(34) $$\sum_{j=k-\bar{N}+1}^{k}tr\left\{{\bar{H}}_{j}^{T}\left[{\bar{P}}_{\bar{z}\bar{z},j\left|j-1\right.}^{-1}-{\bar{P}}_{\bar{z}\bar{z},j\left|j-1\right.}^{-1}{\tilde{\tilde{z}}}_{j}{\tilde{\tilde{z}}}_{j}^{T}{\bar{P}}_{\bar{z}\bar{z},j\left|j-1\right.}^{-1}\right]{\bar{H}}_{j}\frac{\partial \bar{Q}}{\partial {\bar{Q}}_{il}}\right\}=0$$

The measurement update process of the CKF is performed in an identical way to the KF, so the Kalman gain can be rewritten as

(35) $${\bar{K}}_{k}={\bar{P}}_{k\left|k-1\right.}{\bar{H}}_{k}^{T}{\bar{P}}_{\bar{z}\bar{z},k\left|k-1\right.}^{-1}$$

Utilizing Equation (35), Equation (34) can be re-expressed as follows:

(36) $${\sum_{j=k-\bar{N}+1}^{k}\left\{{\bar{P}}_{j\left|j-1\right.}^{-1}\left[{\bar{K}}_{j}{\bar{H}}_{j}{\bar{P}}_{j\left|j-1\right.}-{\bar{K}}_{j}{\tilde{\tilde{z}}}_{j}{\tilde{\tilde{z}}}_{j}^{T}{\bar{K}}_{j}^{T}\right]{\bar{P}}_{j\left|j-1\right.}^{-1}\right\}}_{li}=0$$

Assuming the filtering process within the estimation window is stable, ${\bar{P}}_{j\left|j-1\right.}$ can be approximated as a fixed value, and Equation (36) can be rewritten as

(37) $${\left\{{\bar{P}}_{k\left|k-1\right.}^{-1}\sum_{j=k-\bar{N}+1}^{k}\left[{\bar{K}}_{j}{\bar{H}}_{j}{\bar{P}}_{j\left|j-1\right.}-{\bar{K}}_{j}{\tilde{\tilde{z}}}_{j}{\tilde{\tilde{z}}}_{j}^{T}{\bar{K}}_{j}^{T}\right]{\bar{P}}_{k\left|k-1\right.}^{-1}\right\}}_{li}=0$$

And when Equation (38) is satisfied, Equation (37) is always true.

(38) $$\sum_{j=k-\bar{N}+1}^{k}\left[{\bar{K}}_{j}{\bar{H}}_{j}{\bar{P}}_{j\left|j-1\right.}-{\bar{K}}_{j}{\tilde{\tilde{z}}}_{j}{\tilde{\tilde{z}}}_{j}^{T}{\bar{K}}_{j}^{T}\right]=0$$

According to the Kalman filtering process, it is known that the following equations hold:

(39) $${\bar{K}}_{k}{\tilde{\tilde{z}}}_{k}={\overset{︹}{x}}_{k\left|k\right.}-{\overset{︹}{x}}_{k\left|k-1\right.}$$

(40) $${\bar{P}}_{k\left|k-1\right.}-{\bar{P}}_{k\left|k\right.}={\bar{K}}_{k}{\bar{H}}_{k}{\bar{P}}_{k\left|k-1\right.}$$

(41) $${\bar{P}}_{k\left|k-1\right.}=\sum_{i=1}^{2\bar{n}}{\bar{\omega }}_{i}\left[f({\bar{X}}_{i,k-1\left|k-1\right.})-{\overset{︹}{x}}_{k\left|k-1\right.}\right]{\left[f({\bar{X}}_{i,k-1\left|k-1\right.})-{\overset{︹}{x}}_{k\left|k-1\right.}\right]}^{T}+\bar{Q}$$

Substituting Equations (39)–(41) into Equation (38), the ML estimate of the covariance matrix $\bar{Q}$ is approximated as

(42) $${\bar{Q}}_{ML}=\frac{1}{\bar{N}}\sum_{j=k-\bar{N}+1}^{k}\left[\begin{array}{l} {\bar{P}}_{j\left|j\right.}+({\overset{︹}{x}}_{j\left|j\right.}-{\overset{︹}{x}}_{j\left|j-1\right.}){\left({\overset{︹}{x}}_{j\left|j\right.}-{\overset{︹}{x}}_{j\left|j-1\right.}\right)}^{T}-\\ \sum_{i=1}^{2\bar{n}}{\bar{\omega }}_{i}\left\{f({\bar{X}}_{i,j-1\left|j-1\right.})-{\overset{︹}{x}}_{j\left|j-1\right.}\right\}{\left\{f({\bar{X}}_{i,j-1\left|j-1\right.})-{\overset{︹}{x}}_{j\left|j-1\right.}\right\}}^{T} \end{array}\right]$$

The performance of the maximum likelihood (ML)-based process noise covariance estimator is highly dependent on the size of the fixed-length memory window, $\bar{N}$, whose selection represents a critical trade-off among estimation stability, tracking capability for time-varying noise, and computational efficiency. To ensure estimation stability, the window must be sufficiently large to encompass enough innovation samples for statistical reliability, as an excessively small $\bar{N}$ (e.g., $\bar{N}<5$) may lead to high-variance estimates sensitive to outliers and potential filter instability. Conversely, an overly large $\bar{N}$ (e.g., $\bar{N}>20$) diminishes tracking capability by oversmoothing the estimate and introducing lag in responding to abrupt changes in noise statistics or system dynamics, thereby degrading filtering accuracy during transitions. Computational efficiency also imposes a practical constraint, as complexity scales linearly with $\bar{N}$, favoring a smaller window when possible. Synthesizing these criteria, the typical value for $\bar{N}$ ranges from 5 to 20; for this study, $\bar{N}=10$ was selected via pre-trials ($\bar{N}=\{5,10,15,20\}$) for a navigation system with state dimension $\bar{n}=17$, as it optimally balanced stability and tracking performance, a result validated by the convergence analysis in Section 4.2. While the optimal $\bar{N}$ may vary with application-specific dynamics, future work could explore adaptive strategies for online adjustment to further enhance performance across operational phases.

Next, we use the estimated process noise covariance, the predicted cubature point error matrix from the likelihood function, and the posterior cubature point error obtained from the linear transformation of the model prediction residual to approximate the likelihood function and update the posterior cubature point, thereby constructing a modified cubature point update framework. Define the prior cubature point error matrix ${\tilde{\tilde{X}}}_{k\left|k\right.}^{-}$, the posterior cubature point error matrix ${\tilde{\tilde{X}}}_{k\left|k\right.}^{+}$, and the weight matrix $\hat{W}$ as

(43) $${\tilde{\tilde{X}}}_{k\left|k\right.}^{-}=\left[{\bar{X}}_{1,k\left|k-1\right.}^{*}-{\overset{︹}{x}}_{k\left|k-1\right.},{\bar{X}}_{2,k\left|k-1\right.}^{*}-{\overset{︹}{x}}_{k\left|k-1\right.},\cdots ,{\bar{X}}_{2\bar{n},k\left|k-1\right.}^{*}-{\overset{︹}{x}}_{k\left|k-1\right.}\right]$$

(44) $${\tilde{\tilde{X}}}_{k\left|k\right.}^{+}=\left[{\bar{X}}_{1,k\left|k\right.}-{\overset{︹}{x}}_{k\left|k\right.},{\bar{X}}_{2,k\left|k\right.}-{\overset{︹}{x}}_{k\left|k\right.},\cdots ,{\bar{X}}_{2\bar{n},k\left|k\right.}-{\overset{︹}{x}}_{k\left|k\right.}\right]$$

(45) $$\hat{W}=diag({\hat{\omega }}_{1},{\hat{\omega }}_{2},\cdots ,{\hat{\omega }}_{2\bar{n}})$$

Assuming that ${\tilde{\tilde{X}}}_{k\left|k\right.}^{+}$ can be represented by ${\tilde{\tilde{X}}}_{k\left|k\right.}^{+}={\bar{\gamma }}_{k}{\tilde{\tilde{X}}}_{k\left|k\right.}^{-}$, the following constraint equation holds:

(46) $$\left\{\begin{array}{l} {\tilde{\tilde{X}}}_{k\left|k\right.}^{-}\hat{\omega }=0\\ {\tilde{\tilde{X}}}_{k\left|k\right.}^{+}\hat{\omega }=0 \end{array}\right.$$

(47) $${\tilde{\tilde{X}}}_{k\left|k\right.}^{-}\hat{W}{\left({\tilde{\tilde{X}}}_{k\left|k\right.}^{-}\right)}^{T}={\bar{P}}_{k\left|k-1\right.}-{\bar{Q}}_{ML}$$

(48) $${\tilde{\tilde{X}}}_{k\left|k\right.}^{+}\hat{W}{\left({\tilde{\tilde{X}}}_{k\left|k\right.}^{+}\right)}^{T}={\bar{P}}_{k\left|k\right.}-\Delta {\bar{R}}_{k}$$

where $\hat{\omega }={\left[{\hat{\omega }}_{1},{\hat{\omega }}_{2},\cdots ,{\hat{\omega }}_{2\bar{n}}\right]}^{T}$, $\Delta {\bar{R}}_{k}$ is used to account for approximate uncertainty and $\Delta {\bar{R}}_{k}={\bar{\Lambda }}_{k}{\bar{K}}_{k}{\bar{R}}_{k}{\bar{K}}_{k}^{T}$, and ${\bar{\Lambda }}_{k}$ represents the scale matrix.

The matrix $\Delta {\bar{R}}_{k}$ is introduced to account for the approximation uncertainty inherent in the statistical linearization process of the nonlinear measurement update. It compensates for errors arising from the linearization of h(⋅) and any unmodeled dynamics not captured by the nominal measurement noise covariance $\Delta {\bar{R}}_{k}$.

The scaling matrix ${\bar{\Lambda }}_{k}$ is a positive definite diagonal matrix designed to regulate the influence of $\Delta {\bar{R}}_{k}$ based on the observability of different states. In tightly coupled INS/GNSS integration, states such as position, velocity, and receiver clock error are highly observable through GNSS measurements. In contrast, states like attitude errors and IMU sensor biases are not directly observable; their estimation relies on coupling through the system dynamics. To ensure filter stability and robustness, the diagonal elements ${\bar{\Lambda }}_{ii}$ of ${\bar{\Lambda }}_{k}$ are set as follows: ${\bar{\Lambda }}_{ii}=0$ for states with low observability (e.g., attitude, biases), which prevents spurious inflation of their posterior covariance from measurement approximation errors; and ${\bar{\Lambda }}_{ii}=1$ for states with high observability (e.g., position, velocity, clock), allowing the measurement update to exert its full corrective influence while still accounting for approximation uncertainty. This design ensures that the posterior covariance inflation is focused only on states that can be reliably corrected by measurements, thereby enhancing the filter’s robustness against model uncertainties and observation losses. The choice of ${\bar{\Lambda }}_{k}$ directly impacts the posterior cubature points via Equation (50), ensuring their spread accurately reflects the true state uncertainty.

Under the constraint of Equations (47) and (48), we obtain

(49) $${\bar{\gamma }}_{k}={\bar{M}}_{k}^{+}{\left({\bar{M}}_{k}^{-}\right)}^{-1}$$

where ${\bar{M}}_{k}^{+}=chol({\bar{P}}_{k\left|k\right.}-\Delta {\bar{R}}_{k})$, ${\bar{M}}_{k}^{-}=chol({\bar{P}}_{k\left|k-1\right.}-{\bar{Q}}_{ML})$.

Finally, according to Equations (43), (44), and (49), the cubature points for instance k+1 can be written as follows:

(50) $$\begin{array}{l} {\bar{X}}_{i,k\left|k\right.}={\overset{︹}{x}}_{k\left|k\right.}+{\tilde{\tilde{X}}}_{i,k\left|k\right.}^{+}\\ ={\overset{︹}{x}}_{k\left|k\right.}+{\bar{M}}_{k}^{+}{\left({\bar{M}}_{k}^{-}\right)}^{-1}({\bar{X}}_{i,k\left|k-1\right.}^{*}-{\overset{︹}{x}}_{k\left|k-1\right.}) \end{array}$$

It is worth noting that in the derivation of the ML-based covariance estimation, we assume a constant dimension of the measurement vector ${\bar{z}}_{k}$ within the estimation window. In practice, if the number of available GNSS satellites changes, appropriate data completion or dimension normalization techniques should be applied to maintain consistency. Future work will address adaptive dimension handling in the ML estimation framework.

By introducing the estimated process noise covariance and modified cubature point update strategy into the cubature Kalman filter framework, an improved CKF is developed, which is named ICKF. The ICKF is summarized as follows:

(1) Initialize the Cubature Points

Given the state estimate ${\overset{︹}{x}}_{k-1\left|k-1\right.}$, error covariance ${\bar{P}}_{k-1\left|k-1\right.}$, process noise covariance ${\bar{Q}}_{ML}$, and measurement noise covariance ${\bar{R}}_{k}$, initialize cubature points ${\bar{X}}_{i,k-1\left|k-1\right.}$ by CKF.

(2) Time Update

Calculate ${\overset{︹}{x}}_{k\left|k-1\right.}$ and ${\bar{P}}_{k\left|k-1\right.}$ by

(51) $${\overset{︹}{x}}_{k\left|k-1\right.}=\sum_{i=1}^{2\bar{n}}{\bar{\omega }}_{i}f({\bar{X}}_{i,k-1\left|k-1\right.})$$

(52) $${\bar{P}}_{k\left|k-1\right.}=\sum_{i=1}^{2\bar{n}}{\bar{\omega }}_{i}\left[f({\bar{X}}_{i,k-1\left|k-1\right.})-{\overset{︹}{x}}_{k\left|k-1\right.}\right]{\left[f({\bar{X}}_{i,k-1\left|k-1\right.})-{\overset{︹}{x}}_{k\left|k-1\right.}\right]}^{T}+{\bar{Q}}_{ML}$$

Compute the predicted cubature point error matrix ${\tilde{\tilde{X}}}_{s,k\left|k\right.}^{-}$ and update ${\tilde{\tilde{X}}}_{k\left|k\right.}^{-}$ by

(53) $${\tilde{\tilde{X}}}_{s,k\left|k\right.}^{-}=\left[f({\bar{X}}_{1,k-1\left|k-1\right.})-{\overset{︹}{x}}_{k\left|k-1\right.},f({\bar{X}}_{2,k-1\left|k-1\right.})-{\overset{︹}{x}}_{k\left|k-1\right.},\cdots ,f({\bar{X}}_{2\bar{n},k-1\left|k-1\right.})-{\overset{︹}{x}}_{k\left|k-1\right.}\right]$$

(54) $${\tilde{\tilde{X}}}_{k\left|k\right.}^{-}=chol({\bar{P}}_{k\left|k-1\right.}){\left[chol({\bar{P}}_{k\left|k-1\right.}-{\bar{Q}}_{ML})\right]}^{-1}{\tilde{\tilde{X}}}_{s,k\left|k\right.}^{-}$$

Generate cubature points by Equation (55).

(55) $${\bar{X}}_{i,k\left|k-1\right.}={\overset{︹}{x}}_{k\left|k-1\right.}+{\tilde{\tilde{X}}}_{i,k\left|k\right.}^{-}$$

(3) Measurement Update

Calculate ${\overset{︹}{z}}_{k\left|k-1\right.}$, ${\bar{P}}_{\bar{z}\bar{z},k\left|k-1\right.}$ and ${\bar{P}}_{\bar{x}\bar{z},k\left|k-1\right.}$ by

(56) $${\overset{︹}{z}}_{k\left|k-1\right.}=\sum_{i=1}^{2\bar{n}}{\bar{\omega }}_{i}h({\bar{X}}_{i,k\left|k-1\right.})$$

(57) $${\bar{P}}_{\bar{z}\bar{z},k\left|k-1\right.}=\sum_{i=1}^{2\bar{n}}{\bar{\omega }}_{i}\left[h({\bar{X}}_{i,k\left|k-1\right.})-{\overset{︹}{z}}_{k\left|k-1\right.}\right]{\left[h({\bar{X}}_{i,k\left|k-1\right.})-{\overset{︹}{z}}_{k\left|k-1\right.}\right]}^{T}+{\bar{R}}_{k}$$

(58) $${\bar{P}}_{\bar{x}\bar{z},k\left|k-1\right.}=\sum_{i=1}^{2\bar{n}}{\bar{\omega }}_{i}\left[f({\bar{X}}_{i,k-1\left|k-1\right.})-{\overset{︹}{x}}_{k\left|k-1\right.}\right]{\left[h({\bar{X}}_{i,k\left|k-1\right.})-{\overset{︹}{z}}_{k\left|k-1\right.}\right]}^{T}$$

Update ${\overset{︹}{x}}_{k\left|k\right.}$ and ${\bar{P}}_{k\left|k\right.}$ by Equations (20) and (21).

(4) Cubature Points Update and Process Noise Covariance Update

Calculate ${\tilde{\tilde{X}}}_{k\left|k\right.}^{+}$ by ${\tilde{\tilde{X}}}_{k\left|k\right.}^{+}={\bar{\gamma }}_{k}{\tilde{\tilde{X}}}_{k\left|k\right.}^{-}$, then the cubature points ${\bar{X}}_{i,k\left|k\right.}$ and process noise covariance ${\bar{Q}}_{ML}$ for instant k+1 are updated by Equations (50) and (42), respectively.

**Figure 1:**
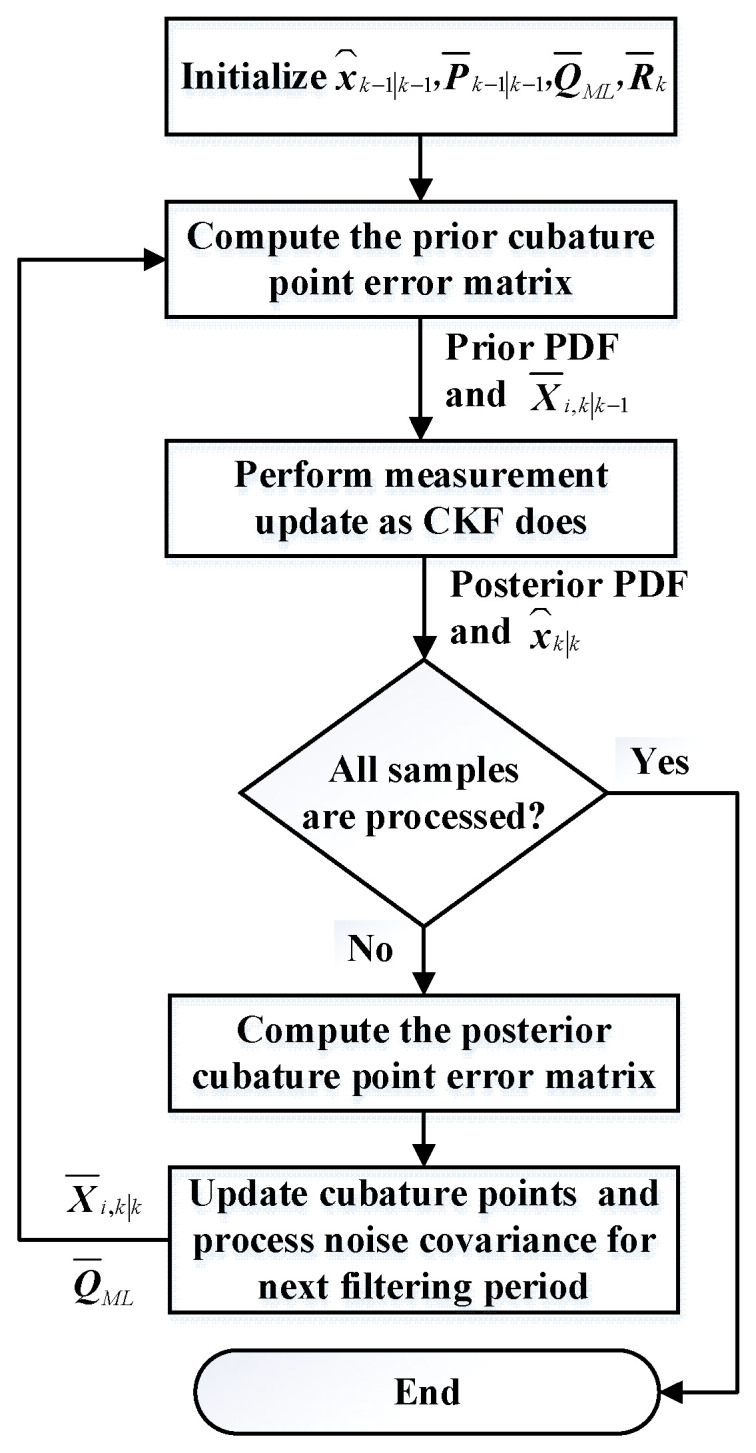
Flowchart of ICKF.

Finally, return to step (2) for the next filtering. Based on the above process, the flowchart of ICKF is shown in Figure 1.

**Remark** **5.** *The implementation of the ICKF involves incorporating an estimation window with a fixed-length memory into the ML estimator. This allows for the integration of new observations while gradually discarding older data. As a result, the ICKF algorithm can estimate and update the process noise covariance more effectively, and incorporate the process noise covariance into the CKF recursive framework based on the posterior cubature point error matrix. Additionally, when observations are missing, no measurement innovation enters the measurement update phase of the filter; instead, the filter operates in prediction mode, and the cubature point error retains the uncertainty from the last measurement update. Therefore, the state prediction accuracy based on ICKF is higher, which slows down the increasing trend of the covariance matrix.*

### 3.2. Stability of the Proposed Algorithm

In this section, we prove the stability of the ICKF.

First, we define the error vectors

(59) $${\tilde{\tilde{x}}}_{k+1}={\bar{x}}_{k+1}-{\overset{︹}{x}}_{k+1\left|k+1\right.}$$

(60) $${\tilde{\tilde{x}}}_{k+1\left|k\right.}={\bar{x}}_{k+1}-{\overset{︹}{x}}_{k+1\left|k\right.}$$

(61) $${\tilde{\tilde{z}}}_{k+1}={\bar{z}}_{k+1}-{\overset{︹}{z}}_{k+1\left|k\right.}$$

By first-order linearization of the model error, we have

(62) $${\tilde{\tilde{x}}}_{k+1\left|k\right.}={\bar{\alpha }}_{k}{\bar{F}}_{k}{\tilde{\tilde{x}}}_{k\left|k-1\right.}-{\bar{\alpha }}_{k}{\bar{F}}_{k}{\bar{K}}_{k}{\tilde{\tilde{z}}}_{k}+{\bar{w}}_{k}$$

(63) $${\tilde{\tilde{z}}}_{k+1}={\bar{\beta }}_{k+1}^{*}{\bar{H}}_{k+1}{\tilde{\tilde{x}}}_{k+1\left|k\right.}+{\bar{v}}_{k}$$

(64) $${\bar{P}}_{k+1\left|k\right.}=\left[{\bar{\alpha }}_{k}{\bar{F}}_{k}(I-{\bar{K}}_{k}{\bar{\beta }}_{k}^{*}{\bar{H}}_{k})\right]{\bar{P}}_{k\left|k-1\right.}{\left[{\bar{\alpha }}_{k}{\bar{F}}_{k}(I-{\bar{K}}_{k}{\bar{\beta }}_{k}^{*}{\bar{H}}_{k})\right]}^{T}+{\bar{Q}}_{k}^{*}$$

where I represents the identity matrix. ${\bar{\alpha }}_{k}=diag({\bar{\alpha }}_{1,k},{\bar{\alpha }}_{2,k},\cdots ,{\bar{\alpha }}_{\bar{n},k})$ represents a diagonal scaling matrix used to bound the linearization error of the system model, where ${\bar{\alpha }}_{i,k}(i=1,2,\ldots \bar{n})$ are scalar scaling factors. ${\bar{\beta }}_{k}^{*}=diag({\bar{\beta }}_{1,k}^{*},{\bar{\beta }}_{2,k}^{*},\cdots ,{\bar{\beta }}_{\bar{m},k}^{*})$ represents a diagonal scaling matrix used to bound the linearization error of the measurement model, where ${\bar{\beta }}_{i,k}^{*}(i=1,2,\ldots \bar{m})$ are scalar scaling factors. ${\bar{Q}}_{k}^{*}={\bar{\alpha }}_{k}{\bar{F}}_{k}{\bar{K}}_{k}{\bar{R}}_{k}{\left({\bar{\alpha }}_{k}{\bar{F}}_{k}{\bar{K}}_{k}\right)}^{T}+\Delta {\bar{P}}_{k+1}+\delta {\bar{P}}_{k+1}+{\bar{Q}}_{ML}$ represents a fictitious process noise covariance matrix constructed for the purpose of the stability proof, encompassing all the error terms. $\Delta {\bar{P}}_{k+1}$ represents the difference when the mean is removed, $\delta {\bar{P}}_{k+1}$ denotes the discrepancy between the actual covariance and the estimated covariance.

We analyze the nonlinear system described by Equations (1) and (2) and apply the ICKF algorithm. We assume that the following conditions hold:

(1) There are positive real constants $f_{low}$, $h_{low}$, $\beta_{low}$, $\alpha_{low}$, $\alpha_{up}$, $q_{low}$, $q_{up}$, $q_{low}^{*}$, $r_{up}$, $p_{low}$, and $p_{up}$ that satisfy the following relationship for k≥0:
  (65) $$\begin{array}{l} f_{low}^{2}I\leq {\bar{F}}_{k}{\bar{F}}_{k}^{T},\;h_{low}^{2}I\leq {\bar{H}}_{k}{\bar{H}}_{k}^{T},\beta_{low}I\leq {\bar{\beta }}_{k}^{*}\\ \alpha_{low}I\leq {\bar{\alpha }}_{k}\leq \alpha_{up}I,q_{low}I\leq {\bar{Q}}_{ML}\leq q_{up}I\\ {\bar{R}}_{k}\leq r_{up}I,p_{low}I\leq {\bar{P}}_{k\left|k\right.}\leq p_{up}I,q_{low}^{*}I\leq {\bar{Q}}_{k}^{*} \end{array}$$
(2) There are real constants $f_{up}$, $h_{up}$, and $\beta_{up}$ that satisfy the following boundary conditions:

(66) $$\left\|{\bar{\alpha }}_{k}{\bar{F}}_{k}\right\|\leq f_{up},\left\|{\bar{\beta }}_{k}^{*}{\bar{H}}_{k}\right\|\leq h_{up},\left\|{\bar{\beta }}_{k}^{*}\right\|\leq \beta_{up}$$

and we need to prove $\underset{k\to \infty }{\lim}{\tilde{\tilde{x}}}_{k}=0$.

Let us set $V_{k+1}({\tilde{\tilde{x}}}_{k+1\left|k\right.})={\tilde{\tilde{x}}}_{k+1\left|k\right.}^{T}{\bar{P}}_{k+1\left|k\right.}^{-1}{\tilde{\tilde{x}}}_{k+1\left|k\right.}$. The above conditions and Equation (62) imply that $p_{low}\alpha_{low}^{2}f_{low}^{2}I\leq {\bar{P}}_{k+1\left|k\right.}\leq p_{up}\alpha_{up}^{2}f_{up}^{2}I+q_{up}I$. Then we get

(67) $$\frac{{\left\|{\tilde{\tilde{x}}}_{k+1\left|k\right.}\right\|}^{2}}{p_{up}\alpha_{up}^{2}f_{up}^{2}I+q_{up}I}\leq V_{k+1}({\tilde{\tilde{x}}}_{k+1\left|k\right.})\leq \frac{{\left\|{\tilde{\tilde{x}}}_{k+1\left|k\right.}\right\|}^{2}}{p_{low}\alpha_{low}^{2}f_{low}^{2}I}$$

Taking the conditional expectation, we obtain

(68) $$\begin{array}{l} E\left\{V_{k+1}({\tilde{\tilde{x}}}_{k+1\left|k\right.})|{\tilde{\tilde{x}}}_{k\left|k-1\right.}\right\}\\ ={\tilde{\tilde{x}}}_{k\left|k-1\right.}^{T}{\left[{\bar{\alpha }}_{k}{\bar{F}}_{k}(I-{\bar{K}}_{k}{\bar{\beta }}_{k}^{*}{\bar{H}}_{k})\right]}^{T}{\bar{P}}_{k+1\left|k\right.}^{-1}\left[{\bar{\alpha }}_{k}{\bar{F}}_{k}(I-{\bar{K}}_{k}{\bar{\beta }}_{k}^{*}{\bar{H}}_{k})\right]{\tilde{\tilde{x}}}_{k\left|k-1\right.}\\ +E\left\{{\bar{v}}_{k}^{T}{\left[{\bar{\alpha }}_{k}{\bar{F}}_{k}{\bar{K}}_{k}\right]}^{T}{\bar{P}}_{k+1\left|k\right.}^{-1}\left[{\bar{\alpha }}_{k}{\bar{F}}_{k}{\bar{K}}_{k}\right]{\bar{v}}_{k}+{\bar{w}}_{k}^{T}{\bar{P}}_{k+1\left|k\right.}^{-1}{\bar{w}}_{k}\left|{\tilde{\tilde{x}}}_{k\left|k-1\right.}\right.\right\} \end{array}$$

According to Equation (64), we obtain

(69) $$\begin{array}{l} {\bar{P}}_{k+1\left|k\right.}=\left[{\bar{\alpha }}_{k}{\bar{F}}_{k}(I-{\bar{K}}_{k}{\bar{\beta }}_{k}^{*}{\bar{H}}_{k})\right]\left\{{\bar{P}}_{k\left|k-1\right.}+{\left[{\bar{\alpha }}_{k}{\bar{F}}_{k}(I-{\bar{K}}_{k}{\bar{\beta }}_{k}^{*}{\bar{H}}_{k})\right]}^{-1}\right.\\ \times \left.{\bar{Q}}_{k}^{*}{\left[{\bar{\alpha }}_{k}{\bar{F}}_{k}(I-{\bar{K}}_{k}{\bar{\beta }}_{k}^{*}{\bar{H}}_{k})\right]}^{-T}\right\}{\left[{\bar{\alpha }}_{k}{\bar{F}}_{k}(I-{\bar{K}}_{k}{\bar{\beta }}_{k}^{*}{\bar{H}}_{k})\right]}^{T} \end{array}$$

On the basis of Equation (66), we have [38]

(70) $$\begin{array}{l} \left\|{\bar{\alpha }}_{k}{\bar{F}}_{k}(I-{\bar{K}}_{k}{\bar{\beta }}_{k}^{*}{\bar{H}}_{k}){\bar{Q}}_{k}^{*-T}{\left[{\bar{\alpha }}_{k}{\bar{F}}_{k}(I-{\bar{K}}_{k}{\bar{\beta }}_{k}^{*}{\bar{H}}_{k})\right]}^{T}\right\|\\ \leq {\left(\alpha_{up}f_{up}+\alpha_{up}f_{up}{\bar{K}}^{*}\beta_{up}h_{up}\right)}^{2}\left\|{\bar{Q}}_{k}^{*-T}\right\| \end{array}$$

where ${\bar{K}}^{*}$ represents the upper bound of ${\bar{K}}_{k}$. According to Equations (65) and (70), we obtain

(71) $$\begin{array}{l} {\left[{\bar{\alpha }}_{k}{\bar{F}}_{k}(I-{\bar{K}}_{k}{\bar{\beta }}_{k}^{*}{\bar{H}}_{k})\right]}^{-1}{\bar{Q}}_{k}^{*}{\left[{\bar{\alpha }}_{k}{\bar{F}}_{k}(I-{\bar{K}}_{k}{\bar{\beta }}_{k}^{*}{\bar{H}}_{k})\right]}^{-T}\\ \geq \frac{q_{low}^{*}I}{{\left(\alpha_{up}f_{up}+\alpha_{up}f_{up}{\bar{K}}^{*}\beta_{up}h_{up}\right)}^{2}} \end{array}$$

Substituting Equation (71) into Equation (69), we have

(72) $$\begin{array}{l} {\left[{\bar{\alpha }}_{k}{\bar{F}}_{k}(I-{\bar{K}}_{k}{\bar{\beta }}_{k}^{*}{\bar{H}}_{k})\right]}^{T}{\bar{P}}_{k+1\left|k\right.}^{-1}\left[{\bar{\alpha }}_{k}{\bar{F}}_{k}(I-{\bar{K}}_{k}{\bar{\beta }}_{k}^{*}{\bar{H}}_{k})\right]\\ \leq {\left[1+\frac{q_{low}^{*}I}{{\left(\alpha_{up}f_{up}+\alpha_{up}f_{up}{\bar{K}}^{*}\beta_{up}h_{up}\right)}^{2}-p_{up}}\right]}^{-1}{\bar{P}}_{k\left|k-1\right.}^{-1} \end{array}$$

Under conditions (1) and (2), we can derive that

(73) $$\begin{array}{l} E\left\{{\bar{v}}_{k}^{T}{\left[{\bar{\alpha }}_{k}{\bar{F}}_{k}{\bar{K}}_{k}\right]}^{T}{\bar{P}}_{k+1\left|k\right.}^{-1}\left[{\bar{\alpha }}_{k}{\bar{F}}_{k}{\bar{K}}_{k}\right]{\bar{v}}_{k}+{\bar{w}}_{k}^{T}{\bar{P}}_{k+1\left|k\right.}^{-1}{\bar{w}}_{k}\left|{\tilde{\tilde{x}}}_{k\left|k-1\right.}\right.\right\}\\ \leq E\left\{\frac{{\bar{K}}^{*2}\alpha_{up}^{2}f_{up}^{2}}{p_{low}}tr({\bar{v}}_{k}^{T}{\bar{v}}_{k})+\frac{1}{p_{low}}tr({\bar{w}}_{k}^{T}{\bar{w}}_{k})\right\}=\mu \end{array}$$

Define ${\left[1+\frac{q_{low}^{*}I}{{\left(\alpha_{up}f_{up}+\alpha_{up}f_{up}{\bar{K}}^{*}\beta_{up}h_{up}\right)}^{2}-p_{up}}\right]}^{-1}=1-\lambda$. By substituting Equations (72) and (73) into Equation (68), we obtain

(74) $$E\left\{V_{k+1}({\tilde{\tilde{x}}}_{k+1\left|k\right.})|{\tilde{\tilde{x}}}_{k\left|k-1\right.}\right\}\leq (1-\lambda )V_{k}({\tilde{\tilde{x}}}_{k\left|k-1\right.})+\mu$$

The boundedness of ${\tilde{\tilde{x}}}_{k+1\left|k\right.}$ in mean square is established through Equations (67) and (74). Consequently, Equation (62) allows us to infer that

(75) $${\left\|{\tilde{\tilde{x}}}_{k}\right\|}^{2}={\left({\bar{\alpha }}_{k}{\bar{F}}_{k}\right)}^{-2}{\left\|{\tilde{\tilde{x}}}_{k+1\left|k\right.}-{\bar{w}}_{k}\right\|}^{2}\leq 2\alpha_{low}^{-2}f_{low}^{-2}\left({\left\|{\tilde{\tilde{x}}}_{k+1\left|k\right.}\right\|}^{2}+{\left\|{\bar{w}}_{k}\right\|}^{2}\right)$$

Taking the expectation of Equation (75), we have

(76) $$E\left\{{\left\|{\tilde{\tilde{x}}}_{k}\right\|}^{2}\right\}\leq 2\alpha_{low}^{-2}f_{low}^{-2}\left(E\left\{{\left\|{\tilde{\tilde{x}}}_{k+1\left|k\right.}\right\|}^{2}\right\}+E\left\{{\left\|{\bar{w}}_{k}\right\|}^{2}\right\}\right)$$

In summary, the mean square bounds of ${\tilde{\tilde{x}}}_{k+1\left|k\right.}$ and ${\bar{w}}_{k}$ are finite, leading to the conclusion that $\underset{k\to \infty }{\lim}{\tilde{\tilde{x}}}_{k}=0$.

### 3.3. Computational Complexity Analysis

In this section, we employ floating-point operations (FLOPs) as a metric to evaluate the computational complexity of ICKF. Drawing upon our previous research [7], we have determined that the overall computational complexity of CKF is $\frac{26}{3}{\bar{n}}^{3}+13{\bar{n}}^{2}+10{\bar{n}}^{2}\bar{m}+8\bar{n}{\bar{m}}^{2}+8\bar{n}\bar{m}+{\bar{m}}^{3}+{\bar{m}}^{2}+\bar{m}$. Note that the main difference between CKF and ICKF is in the process of estimating the process noise covariance and generating cubature points. The FLOPs associated with the generation of cubature points and the estimation of process noise covariance for CKF and ICKF are denoted as $\frac{2}{3}{\bar{n}}^{3}+6{\bar{n}}^{2}$ and $\frac{23+12\bar{N}}{3}{\bar{n}}^{3}+(7\bar{N}+2){\bar{n}}^{2}+2\bar{N}\bar{n}$, respectively. Thus, the computational complexity of ICKF is $\frac{47+12\bar{N}}{3}{\bar{n}}^{3}+(7\bar{N}+9){\bar{n}}^{2}+2\bar{N}\bar{n}+10{\bar{n}}^{2}\bar{m}+8\bar{n}{\bar{m}}^{2}+8\bar{n}\bar{m}+{\bar{m}}^{3}+{\bar{m}}^{2}+\bar{m}\;$.

## 4. Experimental Results and Discussion

In this section, the effectiveness of ICKF is evaluated based on simulations and field tests.

### 4.1. Filter Model of INS/GNSS

The system model of tightly coupled INS/GNSS is selected as the combination of the indirect model of INS error propagation and the clock offset and drift model of the receiver [37]. By choosing the east-north-up as the navigation frame (n-frame), the nonlinear INS state vector error equation can be expressed as

(77) $$\dot{\phi }=C_{\omega }\left[\left(I-C_{n}^{p}\right)\omega_{in}^{n}+\delta \omega_{in}^{n}-C_{b}^{p}\delta \omega_{ib}^{b}\right]$$

(78) $$\delta {\dot{V}}^{n}=\left(I-C_{p}^{n}\right)C_{b}^{p}f_{ib}^{b}+C_{b}^{p}\delta f_{ib}^{b}+\delta V^{n}\times \left(2\omega_{ie}^{n}+\omega_{en}^{n}\right)+V^{n}\times \left(2\delta \omega_{ie}^{n}+\delta \omega_{en}^{n}\right)$$

(79) $$\delta \dot{L}=\frac{\delta V_{N}}{R_{N}+h}$$

(80) $$\delta \dot{\lambda }=\frac{\delta V_{E}}{R_{N}+h}\sec L+\delta L\frac{V_{E}\sec L}{R_{N}+h}\tan L$$

(81) $$\delta \dot{h}=\delta V_{U}$$

where ϕ denotes the attitude vector, δV denotes the velocity error vector in the n-frame, δL, δh, and δλ represent the latitude error vector, the height error vector, and the longitude error vector, respectively. $C_{\omega }$ is the attitude error coefficient transformation (or rotation) matrix; $\omega_{in}^{n}$ is the representation of the angular rate of the navigation frame relative to the inertial frame in the navigation frame and $\delta \omega_{in}^{n}$ is its corresponding error matrix; $C_{n}^{p}$ is the transformation matrix from the ideal navigation frame to the actual navigation frame; $C_{b}^{p}$ is the transformation matrix from body frame to the actual navigation frame; $f_{ib}^{b}$ is the representation of the specific force measurement in the body frame and $\delta f_{ib}^{b}$ is its corresponding error matrix; $\omega_{ie}^{n}$ is the representation of the Earth’s rotation angular rate in the navigation frame and $\delta \omega_{ie}^{n}$ is its corresponding error matrix; $\omega_{en}^{n}$ is the position angular rate in the navigation frame and $\delta \omega_{en}^{n}$ is its corresponding error matrix. The GNSS error state includes the offset $\delta t_{u}$ and drift $\delta t_{ru}$ of the receiver clock, and the corresponding error equation can be expressed as

(82) $$\left\{\begin{array}{l} \delta {\dot{t}}_{u}=\delta t_{ru}+w_{ru}\\ \delta {\dot{t}}_{ru}=-\beta_{tru}\delta t_{ru}+w_{tru} \end{array}\right.$$

where $\beta_{tru}$ is the reciprocal of the correlation time, and $w_{ru}$ and $w_{tru}$ are Gaussian white noise.

The INS/GNSS integrated navigation system constructs measurement equations based on the difference in pseudo-range and pseudo-range rate between the two subsystems. The pseudo-range and pseudo-range rate measurements of INS and GNSS can be represented as

(83) $$\rho_{j}^{\mathrm{INS}}=r_{j}+e_{j1}\delta x+e_{j2}\delta y+e_{j3}\delta z$$

(84) $$\rho_{j}^{\mathrm{GNSS}}=r_{j}+\delta t_{u}+\nu_{\rho j}$$

(85) $${\dot{\rho }}_{j}^{\mathrm{INS}}=e_{j1}\left(\dot{x}-{\dot{x}}_{sj}\right)+e_{j2}\left(\dot{y}-{\dot{y}}_{sj}\right)+e_{j3}\left(\dot{z}-{\dot{z}}_{sj}\right)+e_{j1}\delta \dot{x}+e_{j2}\delta \dot{y}+e_{j3}\delta \dot{z}$$

(86) $${\dot{\rho }}_{j}^{\mathrm{GNSS}}=e_{j1}\delta \dot{x}+e_{j2}\delta \dot{y}+e_{j3}\delta \dot{z}-\delta t_{ru}+\nu_{\dot{\rho }j}$$

where $r_{j}$ is the actual physical distance between the receiver and satellite j; ${\left(\begin{array}{ccc} e_{j1} & e_{j2} & e_{j3} \end{array}\right)}^{T}$ is the directional cosine of the receiver’s line-of-sight direction; $\nu_{\rho j}$ is the receiver’s pseudo-range noise; $\delta \dot{x}$, $\delta \dot{y}$, and $\delta \dot{z}$ are the receiver’s velocity error in the ECEF coordinate frame; $\dot{x}$, $\dot{y}$, and $\dot{z}$ are the changes in velocity, while ${\dot{x}}_{sj}$, ${\dot{y}}_{sj}$, and ${\dot{z}}_{sj}$ are the changes in velocity caused by the receiver’s relative motion to satellite s with respect to satellite j.

### 4.2. Simulations and Analysis

**Figure 2:**
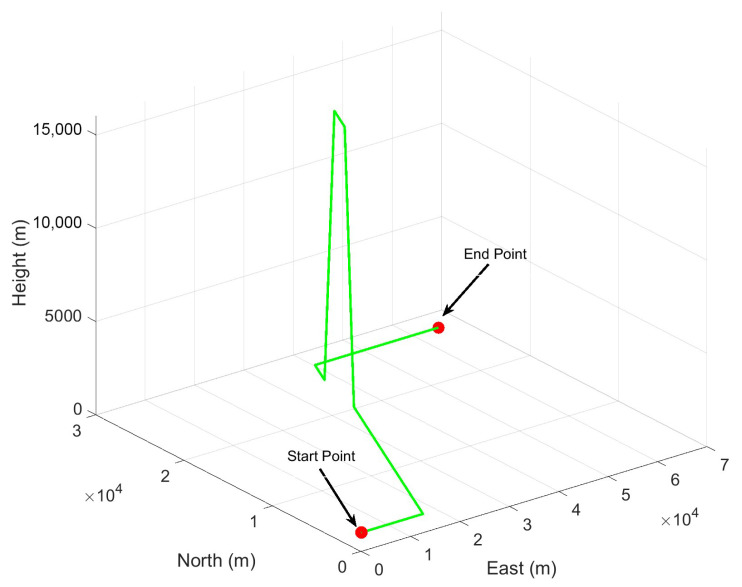
Simulation trajectory of simulation experiment 1.

During the simulation, we designed an aerial vehicle trajectory depicted in Figure 2, lasting 450 s. The specific simulation parameters are given in Table 1. According to the parameters of INS, the initial process noise covariance $\bar{Q}$ and the error covariance matrix ${\bar{P}}_{0\left|0\right.}$ can be set. Although the GNSS observation update rate is 2 Hz, the filter update period is set to 1 s to optimize performance. In the simulations, the trajectory of the aerial vehicle, along with the relevant parameters of INS and GNSS, are artificially configured. Consequently, these parameters enable the determination of the ground truth of the navigation information. To evaluate the filtering performance of ICKF under conditions of process model uncertainty and missing observations, the initial process noise covariance of the filter is set to $20\times \bar{Q}$, and three GNSS-denied scenarios of varying durations are simulated. The size $\bar{N}$ of the fixed-length memory estimation window in ICKF is manually selected through trial, and $\bar{N}$ is set to 10.

**Figure 3:**
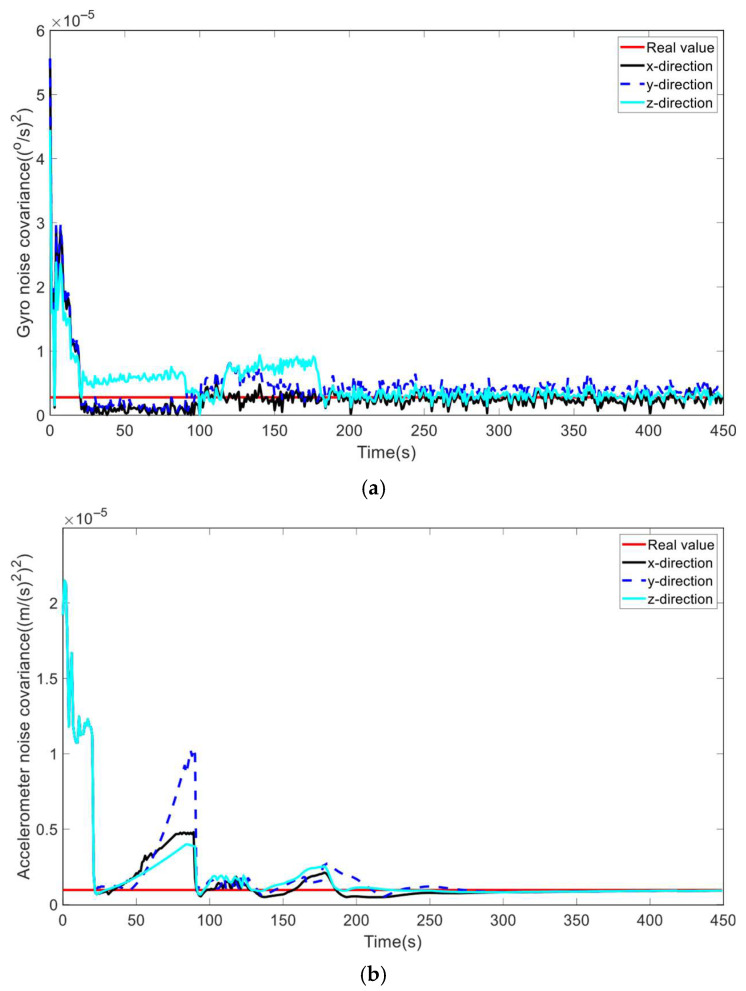
Estimations of the noise covariances. (**a**) Gyro noise covariance; (**b**) accelerometer noise covariance.

Firstly, we conducted experiments to evaluate the performance of ICKF in estimating process noise covariance under the process model uncertainty and GNSS-denied environment, with the GNSS-denied period lasting 60 s from the 30th epoch. The main non-zero elements of the process noise covariance $\bar{Q}$, including the gyroscope noise covariance and accelerometer error covariance, are illustrated in Figure 3, where the horizontal axis represents the simulation time. As depicted in Figure 3, the process noise covariance shows a tendency to diverge during the GNSS-denied phase, but quickly converges, and the estimated gyro and accelerometer noise covariances by the proposed algorithm exhibit remarkable proximity to their actual values following a brief convergence procedure, despite the initial noise covariance matrix being expanded to 20 times its real value. The results indicate that ICKF is capable of accurately estimating the process noise covariance and shows promise in mitigating the impact of uncertainty in the process noise covariance and GNSS denial on the filtering solution of CKF.

**Figure 4:**
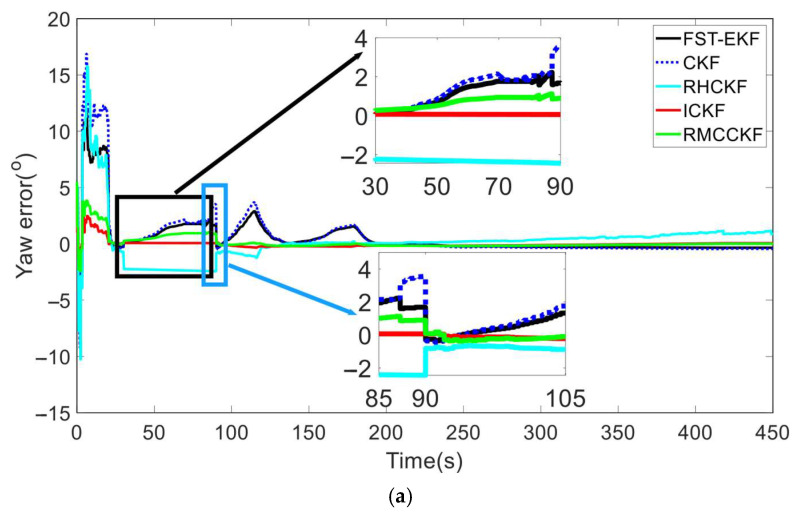

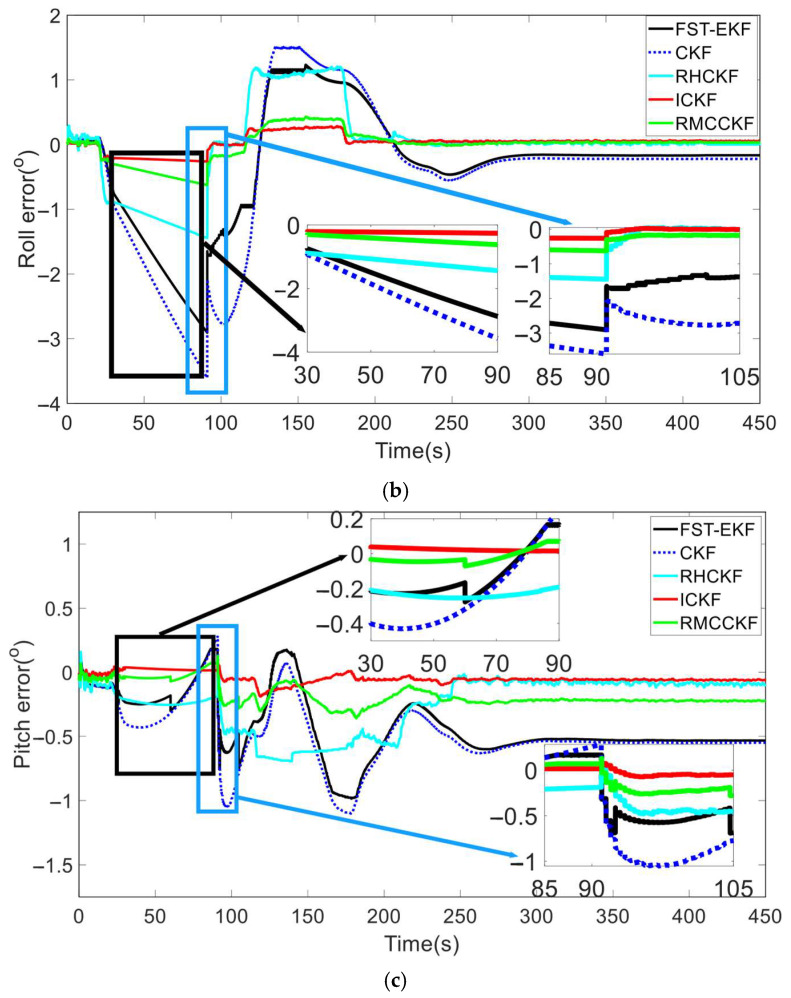
The attitude errors of different algorithms during the simulation process. (**a**) Yaw error; (**b**) roll error; (**c**) pitch error.

**Figure 5:**
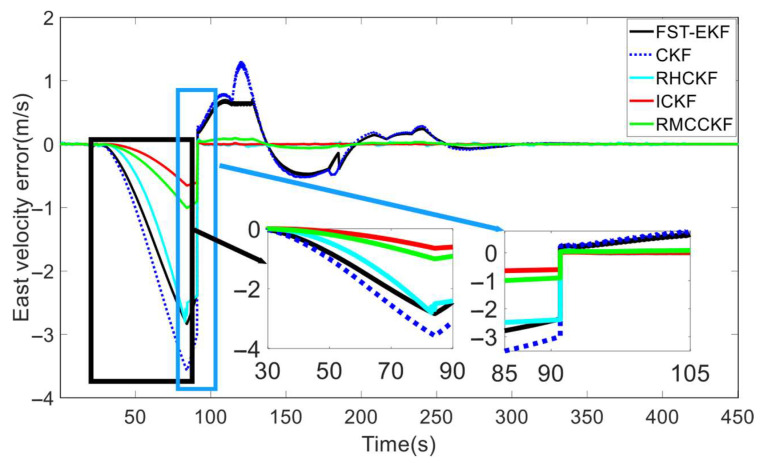
The velocity errors of different algorithms during the simulation process.

Under the aforementioned experimental conditions, we compared the filtering performance of FST-EKF [39], CKF, RHCKF [37], RMCCKF [32], and ICKF, as shown in Figure 4 and Figure 5. Here, the FST-EKF combines the standard EKF with a strong tracking filter, enhanced by fuzzy logic. Fuzzy logic adjusts the fading factor based on the system’s current state, enabling the filter to switch between accurate estimation during stable conditions and quick tracking when sudden changes, such as GNSS signal loss, occur. This adaptive capability allows the FST-EKF to converge back to a stable state after GNSS recovery quickly, outperforming the standard EKF by reducing convergence time and improving estimation accuracy. CKF employs a cubature rule to approximate Gaussian integrals, allowing for more accurate handling of nonlinearities. It utilizes third-degree spherical–radial cubature rules to propagate cubature points through the nonlinear process and measurement functions, providing a more precise approximation of the mean and covariance of the state distribution. While CKF offers improved accuracy for systems with moderate nonlinearities and reduces the errors associated with linearization, it still assumes a fixed process noise covariance. The RHCKF is designed to improve robustness against missing observations and model uncertainty. It introduces the RSUF that mitigates the effects of missing observations and process model uncertainty, maintaining computational efficiency while increasing robustness. However, RHCKF does not dynamically update the process noise covariance. RMCCKF employs the RSUF combined with the maximum correntropy criterion, which adjusts the measurement prediction covariance. The maximum correntropy criterion-based update resizes the measurement prediction covariance, while the RSUF propagates sigma points in a manner that captures the model uncertainties. This design helps to enhance the robustness of the filtering process, particularly under conditions of non-Gaussian noise and missing observations. In Figure 4 and Figure 5, the horizontal axis represents the simulation time. In particular, to better showcase the performance of the algorithms under the simultaneous presence of process model uncertainty and GNSS-denied environment (during the simulation period of 30 s to 90 s), we locally zoom in on Figure 4 and Figure 5. In terms of attitude estimation, Figure 4 highlights the significant advantages of ICKF over other methods. For yaw, roll, and pitch errors, ICKF consistently shows the lowest error levels across the entire simulation period, particularly during GNSS-denied periods. The yaw error, for example, remains minimal and stable even when GNSS signals are lost, contrasting sharply with the large spikes observed for CKF, which suffers from substantial inaccuracies under the same conditions. FST-EKF, RHCKF, and RMCCKF, although more robust than CKF, still exhibit higher errors compared to ICKF, especially during GNSS outages. This demonstrates ICKF’s superior robustness to missing observations and model uncertainties, largely due to its ability to dynamically update the process noise covariance in real time using the ML principle. To more clearly illustrate the dynamic performance of the algorithms at the critical moment of GNSS signal recovery, a zoomed-in inset from 85 s to 105 s has been added to Figure 4. This window specifically captures the key transition period when GNSS signals resume at 90 s. The inset clearly demonstrates that at the moment of signal resumption, ICKF’s attitude errors (yaw, roll, pitch) exhibit the fastest and smoothest convergence. Its error curve turns downward immediately without noticeable overshoot, rapidly returning to a low-error steady state. In contrast, FST-EKF and RMCCKF show some improvement but with slower convergence and slight oscillations; RHCKF exhibits a lagged response; and CKF demonstrates the slowest and most unstable recovery process. This comparison strongly validates that the MUF framework and real-time noise estimation capability of ICKF enable it to most effectively utilize the renewed observational information, rapidly correcting the errors accumulated by the INS during the outage, thereby exhibiting superior stability and rapid recovery performance in the critical transition region. For velocity estimation, Figure 5 further confirms the advantages of ICKF. It maintains significantly lower errors in east velocity, even during prolonged GNSS-denied periods. While CKF experiences considerable error growth due to its inability to adapt to changes in system noise, ICKF effectively mitigates this by continuously updating the process noise covariance, thereby maintaining accuracy. FST-EKF, RHCKF, and RMCCKF perform moderately well, but they still lag behind ICKF, particularly in environments with high process model uncertainty and prolonged GNSS-denied periods. FST-EKF and RMCCKF, which focus on handling GNSS signal loss and non-Gaussian noise, respectively, offer some resilience, yet they cannot achieve the same level of accuracy as ICKF, which is more versatile in handling both noise and uncertainty. Similarly, the zoomed-in inset from 85 s to 105 s in Figure 5 (east velocity error) highlights the divergent convergence behaviors of the algorithms post-recovery. After 90 s, the velocity error of ICKF begins to decrease almost immediately and monotonically, converging towards zero at the fastest rate. This is attributed to its accurate process noise covariance estimation, which leads to a more reasonable calculation of the Kalman gain and higher trust in the new measurements. Other algorithms, particularly CKF and FST-EKF, show error curves that remain elevated or fluctuate for a period after recovery, indicating a longer adjustment time required to handle the transition from pure INS mode to integrated navigation mode. This inset, from the perspective of velocity estimation, further confirms that ICKF possesses superior transient response performance and a shorter convergence time when dealing with critical situations involving abrupt GNSS signal changes.

Overall, the results presented in Figure 4 and Figure 5 clearly demonstrate the superior performance of ICKF in both attitude and velocity estimation under challenging conditions. This is mainly because ICKF performs statistical linear regression (SLR) by introducing the posterior cubature point error matrix without constructing the prior PDF, and the increase in the filter error covariance matrix is bounded by the introduction of the posterior cubature point error matrix from the previous filtering period when GNSS is denied, thus allowing the ICKF to converge quickly to the real state estimate of the carrier at the end of the GNSS-denied period. RHCKF, RMCCKF, and ICKF are all algorithms derived based on the resampling-free sigma-point update framework, and they generate cubature points based on PDF-approximated mean errors, so this class of algorithms has better performance in GNSS-denied environments. The primary distinction between ICKF and RHCKF lies in the former’s capability to estimate and update the process noise covariance using the ML principle, which enhances its robustness to process model uncertainty. In contrast, RHCKF lacks this ability. Compared to ICKF, RMCCKF improves its robustness to non-Gaussian measurement noise by introducing the maximum correntropy criterion but remains sensitive to process model uncertainty. The FST-EKF, by combining fuzzy logic and the extended Kalman filter with strong tracking, can dynamically adjust the filtering gain in real time based on the system’s state error and noise characteristics. When GNSS signals are lost or the process model has uncertainty, the FST-EKF will dynamically adjust the fading factor through fuzzy logic to quickly respond to changes. This dynamic adjustment mechanism enables the algorithm to suppress error accumulation to a certain extent in the face of lost GNSS signals or process model uncertainty, ensuring accurate state estimation. Although FST-EKF addresses some of the limitations of the EKF, it still inherits the EKF’s reliance on nonlinear systems, especially in cases of highly nonlinear systems where the linearization errors are significant, leading to less accurate state estimation. Additionally, in scenarios of prolonged GNSS signal loss, FST-EKF can only rely on the system’s process model for prediction. However, system models usually contain certain errors or uncertainties, which accumulate over time, leading to an increase in the bias of the state estimation. Because the CKF relies on Gaussian assumptions and assumes a fixed process noise covariance without dynamically adjusting for uncertainties, its performance becomes less than ideal under these conditions. Additionally, missing GNSS observations exacerbate this situation, as CKF lacks mechanisms to adapt to such uncertainties.

**Figure 6:**
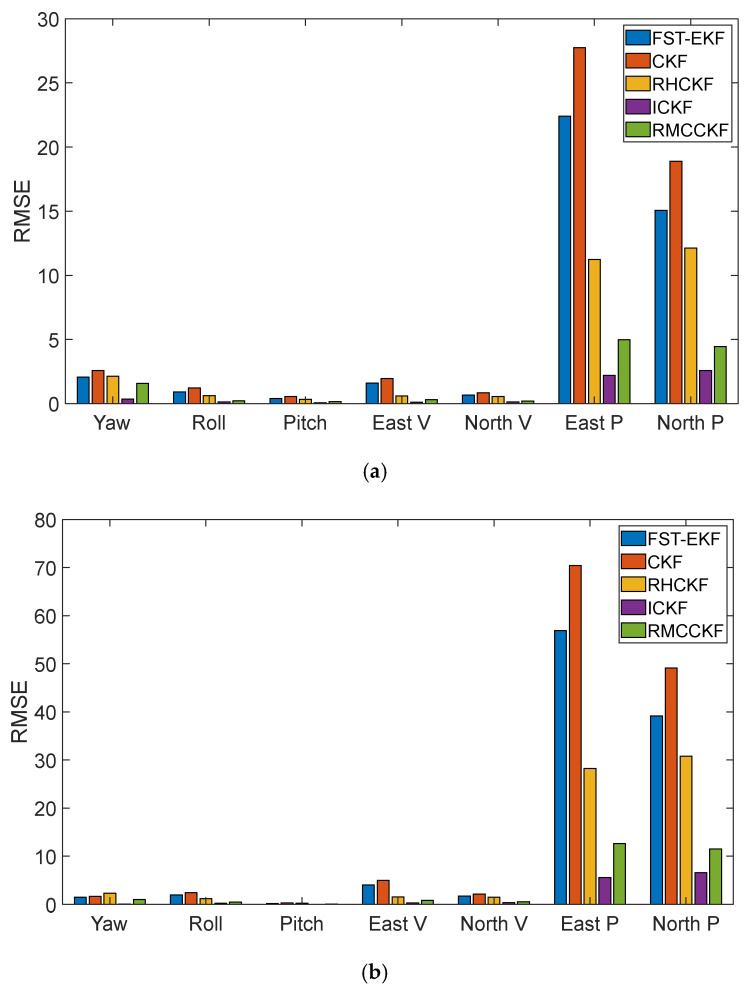
RMSEs of different algorithms. (**a**) RMSEs of different algorithms throughout the entire simulation; (**b**) RMSEs of different algorithms during the period from 30 s to 90 s.

For a more detailed comparison, the root mean square errors (RMSEs) of attitude, velocity (V), and position (P) are shown in Figure 6; the horizontal axis from left to right represents the yaw angle (Yaw), roll angle (Roll), pitch angle (Pitch), east velocity (East V), north velocity (North V), east position (East P), and north position (North P), respectively. Figure 6a,b show the RMSEs of the algorithms throughout the entire simulation and from 30 s to 90 s simulation time (the period with simultaneous process model uncertainty and a GNSS-denied environment), respectively. The figure indicates that velocity and position accuracy deteriorate rapidly during GNSS denial, whereas attitude error is significantly less affected. Since the attitude error is a not directly observable state and its correction information comes from the coupling of the non-diagonal elements of the prediction covariance matrix, the value of ${\bar{P}}_{k\left|k-1\right.}$ is only affected by the cubature point of the system equation transfer and ${\bar{Q}}_{k-1}$ during short-term GNSS observation loss, and the change in ${\bar{P}}_{k\left|k-1\right.}$ will be slow if the change in the system noise can be tracked in real time. In addition, the cubature point information of ICKF is not directly derived from the posterior state estimation of the previous filtering period and the weighting of ${\bar{P}}_{k\left|k-1\right.}$ but is influenced by the cubature point error matrix of the previous filtering period. When the filter stabilizes, the mean error of the state is generally small, making the new cubature point insensitive to the estimation error growth caused by missing measurements; that is, the error of the directly observable state variables in ICKF is smaller than that in FST-EKF, CKF, RHCKF, and RMCCKF.

The vehicle navigation system in urban settings mainly focuses on position accuracy. Therefore, we define ${\mathrm{RMSE}}_{p}$ to evaluate the accuracy of the position.

(87) $${\mathrm{RMSE}}_{p}=\frac{1}{L}\sum_{l=1}^{L}\left({\mathrm{RMSE}}_{e}^{l}+{\mathrm{RMSE}}_{n}^{l}\right)$$

where L denotes the simulation number, and ${\mathrm{RMSE}}_{e}^{l}$ and ${\mathrm{RMSE}}_{n}^{l}$ denote the RMSEs for the east and north positions in the l-th simulation. In addition, another two simulated environments with process model uncertainty and missing observations are simulated, in all of which the filter also uses $20*\bar{Q}$ as the initial process noise covariance, and the GNSS-denied periods last 90 s and 120 s from the 30th epoch, respectively.

For the convenience of analysis, the simulated environments with process model uncertainty and GNSS denial lasting 60 s, 90 s, and 120 s are defined as Environment 1, Environment 2, and Environment 3, respectively. By setting L=500, the ${\mathrm{RMSE}}_{p}$ of the position of different algorithms is presented, where the ${\mathrm{RMSE}}_{p}$ of different algorithms throughout the entire simulation is shown in Table 2, and the ${\mathrm{RMSE}}_{p}$ of different algorithms in the simultaneous presence of process uncertainty and a GNSS-denied environment is shown in Table 3. From the table, we can see that the positioning accuracy of CKF noticeably declines in Environments 1 and 2, whereas the ICKF exhibits the smallest increase in error. In Environment 3, although the ${\mathrm{RMSE}}_{p}$ of the ICKF increases, it remains within an acceptable range. In contrast, the performance of FST-EKF, CKF, RHCKF, and RMCCKF deteriorates significantly. In summary, the clear advantage of ICKF across both tables lies in its ability to dynamically adjust the process noise covariance matrix in real time. This adaptability is critical in maintaining accurate position estimates, especially in GNSS-denied scenarios where unmodeled dynamics and sensor noise can cause significant error accumulation in other filters. CKF is not well-suited for environments with process uncertainty and GNSS-denied conditions. Its reliance on fixed noise models causes rapid error growth, leading to much higher ${\mathrm{RMSE}}_{p}$, particularly in Table 3 where the combined challenges are most severe. While RHCKF and RMCCKF provide better results than CKF, their performance is still limited by their lack of noise adaptability. These methods handle nonlinearities and non-Gaussian noise better but are still prone to error growth in the face of simultaneous process uncertainty and GNSS-denied conditions. FST-EKF has achieved better results than CKF through its strong tracking mechanism and fuzzy logic adaptive gain adjustment. However, it still relies on the local linearization process and has obvious limitations when facing highly nonlinear systems. Therefore, the ${\mathrm{RMSE}}_{p}$ it obtains is still higher than that obtained by RHCKF, RMCCKF, and ICKF.

To evaluate the reliability of the state error covariance matrix estimated by each algorithm, a consistency analysis is conducted. The 3-sigma boundaries are derived from the state error covariance matrix ${\bar{P}}_{k\left|k\right.}$ at each time step, and the actual errors are checked against these boundaries. The percentage of errors falling within the ±3σ boundaries (consistency index) is calculated for the east position, north position, and yaw angle.

Table 4 presents the consistency indices of different algorithms during GNSS outage periods (30–90 s), calculated based on the percentage of actual errors falling within the theoretically derived 3σ boundaries from the state error covariance matrices. The ICKF algorithm demonstrates superior consistency performance with an average index of 96.4%, significantly outperforming other comparative algorithms. This high consistency indicates that ICKF’s state error covariance matrix accurately reflects the actual estimation uncertainty, providing reliable performance assessment in practical applications where ground truth is unavailable. In contrast, CKF shows the lowest consistency (72.6%), suggesting substantial discrepancies between its estimated uncertainty and actual performance.

**Figure 7:**
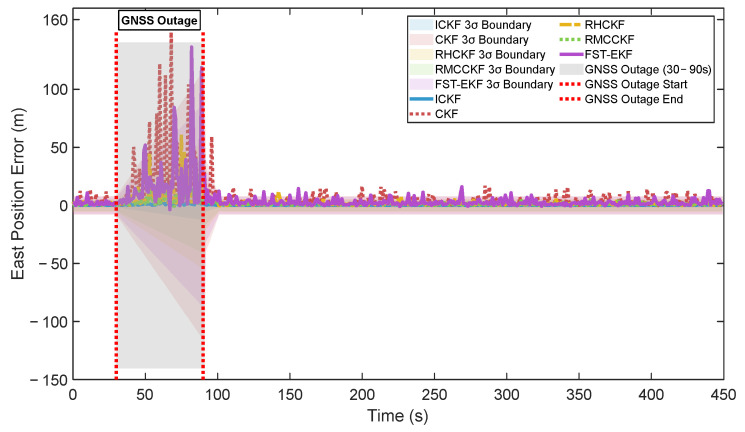
Comparison of east position errors with 3σ boundaries for different algorithms.

**Figure 8:**
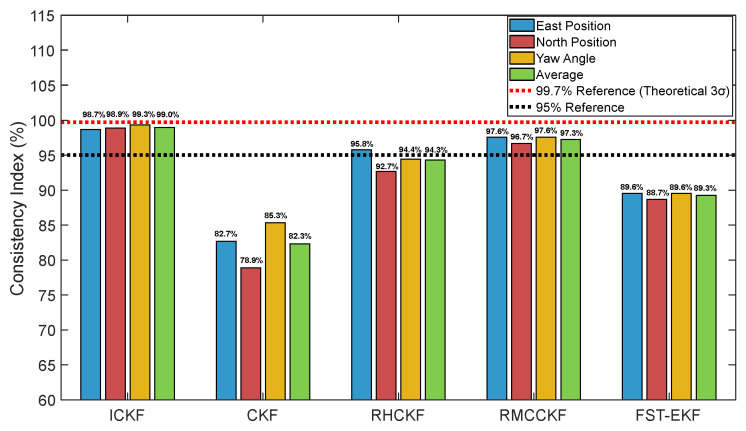
Comparison of consistency indices for different algorithms throughout the simulation.

Figure 7 compares the east position errors of five algorithms with their respective 3σ boundaries derived from state error covariance matrices. The ICKF algorithm demonstrates excellent consistency, with errors predominantly contained within its 3σ boundaries, which are reasonably tight and appropriate. Conversely, CKF exhibits frequent boundary violations and excessively wide uncertainty bounds, indicating poor covariance calibration. Figure 8 visually compares the consistency indices of all algorithms throughout the simulation. ICKF achieves the highest consistency indices for all navigation states, approaching the theoretical optimum of 99.7%. ICKF’s average consistency of 99% demonstrates its exceptional capability in uncertainty quantification, making it particularly valuable for safety-critical applications where reliable performance assessment is essential.

**Figure 9:**
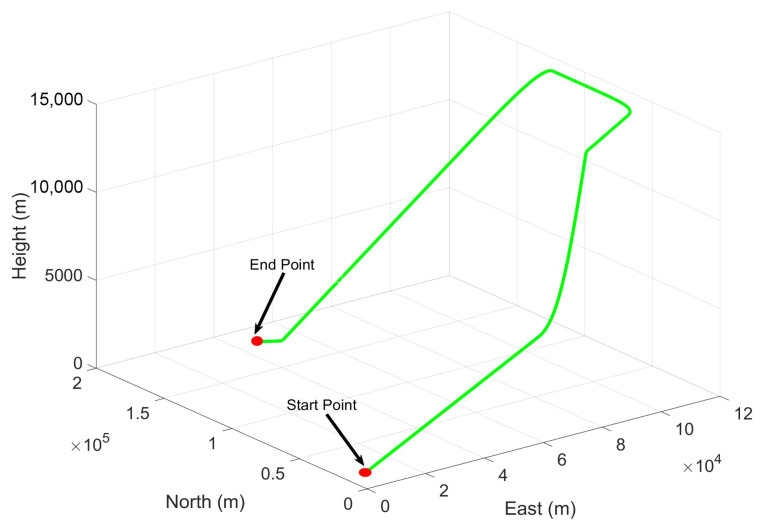
Simulation trajectory of simulation experiment 2.

To further demonstrate the effectiveness of the method proposed in this paper, we designed another flight trajectory of the aerial vehicle for experimentation, as shown in Figure 9. The flight trajectory of the aerial vehicle is divided into four stages: from 0 s to 100 s, the aircraft starts from an initial speed of 300 m/s and an altitude of 1000 m, and rises at a climb angle of approximately 5 degrees. The aircraft continues its flight to the east, progressively increasing its speed while steadily gaining altitude; from 100 s to 200 s, the aircraft’s climb angle increases to 20 degrees and begins to rise more rapidly, with the altitude rapidly increasing. At the same time, the heading angle gradually turns 45 degrees to the right, from due east to northeast; from 200 s to 300 s, the aircraft’s climb angle drops to 0 degrees and enters a level flight state, while the altitude remains unchanged. It continues to turn the heading angle by 90 degrees to the right, causing the aircraft to turn from northeast to due north; from 300 s to 450 s, the aircraft enters the descent phase, the climb angle becomes −10 degrees, and the altitude begins to decrease. The heading angle further turns 45 degrees to the right and then from north to northwest. At nearly 450 s, the aircraft returns to level flight and remains at a stable altitude. In this simulation, all navigation parameters are identical to those set in the first simulation experiment. Additionally, the experimental process includes three different environments, referred to as Environment 1, Environment 2, and Environment 3. Each of these environments involves process uncertainty. The GNSS denial duration for these three environments is as follows: 60 s starting from epoch 50 in Environment 1, 90 s starting from epoch 150 in Environment 2, and 120 s starting from epoch 300 in Environment 3. We present the ${\mathrm{RMSE}}_{p}$ of position for each method under different environments in Table 5. As shown in Table 5, the ${\mathrm{RMSE}}_{p}$ obtained by ICKF is the smallest, followed by RMCCKF, RHCKF, and FST-EKF, while CKF has relatively higher RMSE. Comparing these results with the first simulation experiment, we can conclude that ICKF demonstrates similar effectiveness across different flight paths, indicating that ICKF has good robustness against process model uncertainty and observation loss.

**Figure 10:**
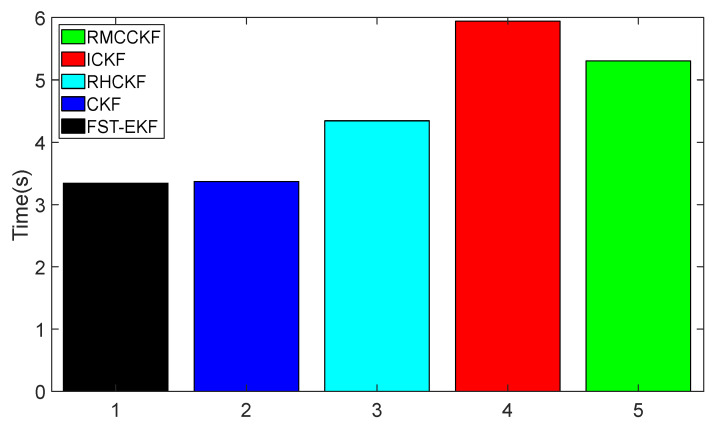
Calculational time comparison.

Additionally, we conducted trials to assess the computational performance of ICKF. The Monte Carlo simulation trials mentioned above were performed using Matlab R2023b programs on a PC with an Intel i7-8565U 1.80 GHz processor and 8 GB of memory. The computational time used by FST-EKF, CKF, RHCKF, ICKF, and RMCCKF to perform one full simulation experiment as described above is illustrated in Figure 10. It can be seen from the figure that the computational time of RHCKF, ICKF, and RMCCKF is higher than that of CKF because they need to adaptively adjust the relevant filtering parameters at each time step during the filtering procedure. Compared to RHCKF and RMCCKF, the filtering process of ICKF involves more inverse and decomposition operations, resulting in higher computational time. In general, ICKF requires more processing time due to its dynamic adjustment of process noise covariance; this additional computational cost is justified by its higher accuracy, especially in process model uncertainty or GNSS-denied scenarios. ICKF balances computational demands and robustness effectively, making it suitable for navigation systems where accuracy and adaptability are priorities, but further improvements are needed for navigation systems with high real-time requirements. The computational cost of FST-EKF is similar to that of the standard EKF, but with the adjustment of fuzzy control and a strong tracking mechanism, the computational cost slightly increases. However, from Figure 10, we can see that it is still slightly lower compared to CKF. On the other hand, RHCKF and RMCCKF have slightly lower computational cost than ICKF, but these filters are less robust against uncertainties in the process model and observation losses when compared to ICKF. Therefore, their performance is limited under conditions where both process uncertainty and observation loss are present simultaneously.

The derivation of the ML estimator for $\bar{Q}$ in Section 3.1 employs the assumption that the filtering process is stable within the estimation window $\bar{N}$, leading to the simplification in Equation (33). To validate this critical assumption and demonstrate the robustness of ICKF even when it is temporarily violated, the following analyses were performed based on the simulation data from Environment 1 (60 s GNSS outage).

**Figure 11:**
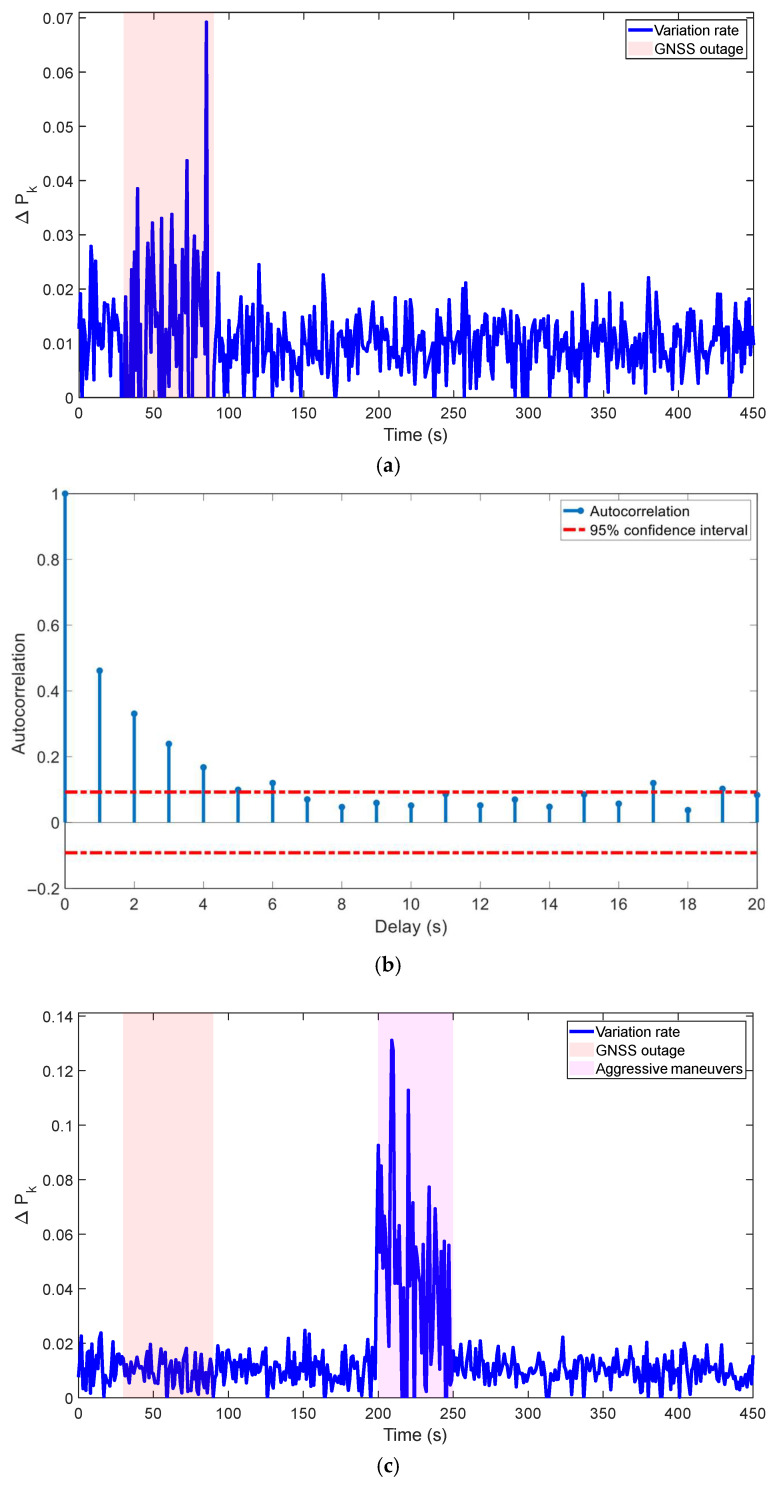
Validation of the stability assumption and robustness of the ICKF. (**a**) Variation rate of the prediction error covariance ($\Delta P_{k}$) during a GNSS outage; (**b**) autocorrelation function (ACF) of the innovation sequence; (**c**) variation rate $\Delta P_{k}$ under aggressive maneuvers and GNSS outage.

(1) Error Covariance Variation Rate: The Frobenius norm of the change in the predicted error covariance matrix between consecutive time steps, defined as $\Delta P_{k}={\left\|{\bar{P}}_{k\left|k-1\right.}-{\bar{P}}_{k-1\left|k-2\right.}\right\|}_{F}$, was computed. As depicted in Figure 11a, $\Delta P_{k}$ remains at a low level (<0.1) for the vast majority of the simulation, indicating that the filtering process is predominantly stable. A moderate increase in $\Delta P_{k}$ is observed during the GNSS outage period (30–90 s), which is expected due to the lack of measurement updates. Crucially, the variation rate remains bounded and does not exhibit divergent behavior.
(2) Whiteness Test of the Innovation Sequence: The stability and optimality of a filter can be assessed by checking if its innovation sequence is a white-noise process. The autocorrelation function (ACF) of the innovation sequence ${\tilde{\tilde{z}}}_{k}$ from the same simulation is plotted in Figure 11b. The results show that almost all autocorrelation values at non-zero lags lie within the 95% confidence bounds (red dashed lines). This confirms that the innovation sequence is essentially uncorrelated, satisfying the whiteness property and indicating that the filter is operating consistently and near-optimally.
(3) Robustness Under Intentional Disturbance: To stress-test the algorithm, an additional period of aggressive maneuver (200–250 s) was introduced into the simulation to intentionally disrupt the filter’s stability. Figure 11c shows the resulting $\Delta P_{k}$. As expected, the covariance variation rate spikes significantly during this maneuver. However, the ICKF algorithm demonstrates strong robustness, as $\Delta P_{k}$ quickly converges back to its nominal low level after the maneuver ends, showcasing the algorithm’s ability to recover from transient periods of instability.

In summary, these validation results confirm that the stability assumption holds for most of the operational time. More importantly, they demonstrate that the ICKF algorithm possesses inherent robustness mechanisms, allowing it to maintain performance and recover stability even when this assumption is temporarily invalidated by challenging conditions like GNSS outages or aggressive dynamics.

The performance of the proposed ICKF algorithm is influenced by several key parameters. To evaluate its robustness and provide practical guidance for implementation, a detailed sensitivity analysis was conducted on the memory window size $\bar{N}$, the initial process noise covariance $\bar{Q}$, and the GNSS measurement noise covariance $\bar{R}$. All analyses were performed under Environment 1 (60 s GNSS outage) using the trajectory from Simulation 1.

**Figure 12:**
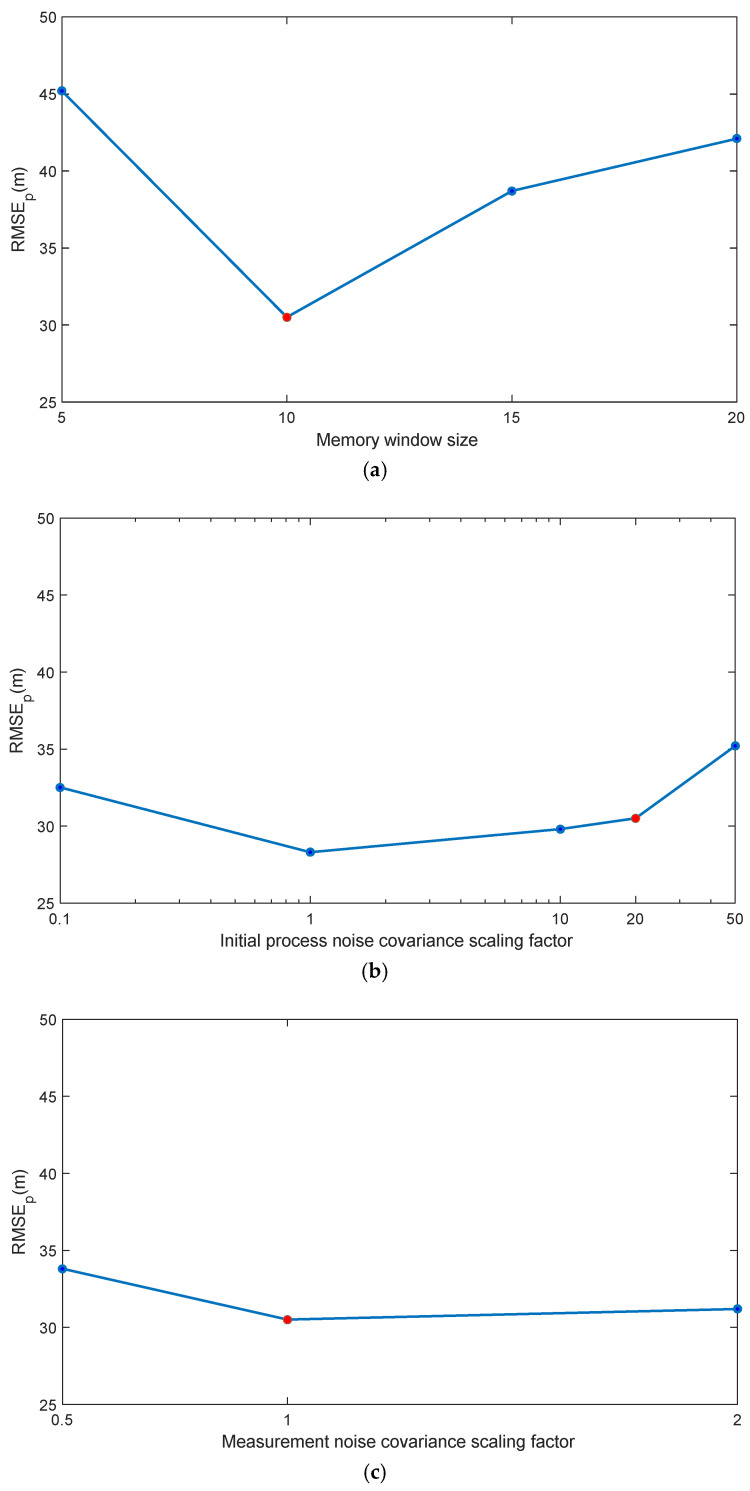
Sensitivity of position ${\mathrm{RMSE}}_{p}$ to key parameters. (**a**) Memory window size $\bar{N}$; (**b**) initial process noise covariance $\bar{Q}$; (**c**) GNSS measurement noise covariance $\bar{R}$.

The memory window size $\bar{N}$ is a critical parameter for the ML estimator, balancing estimation stability and tracking capability for time-varying noise. As previously discussed in Section 3.1 (following Equation (42)), we tested $\bar{N}=\{5,10,15,20\}$. Figure 12a illustrates the relationship between $\bar{N}$ and the position RMSE. A small window ($\bar{N}=5$) provides faster adaptation but results in noisier $\bar{Q}$ estimates and higher RMSE due to insufficient innovation samples. Conversely, a large window ($\bar{N}=20$) oversmooths the $\bar{Q}$ estimate, introducing lag in responding to changes in system dynamics and thus degrading accuracy during the GNSS outage. The value $\bar{N}=10$ was found to optimally balance this trade-off, yielding the lowest RMSE, and was therefore selected for all other experiments.

The initial value of the process noise covariance $\bar{Q}$ is often uncertain in practice. To assess ICKF’s sensitivity to this parameter, we initialized $\bar{Q}$ with values scaled from the nominal $\bar{Q}$: ${\bar{Q}}_{init}=\{0.1,1,10,20,50\}\times \bar{Q}$. The resulting position RMSE values are shown in Figure 12b. The results indicate that the ICKF is highly robust to the initial $\bar{Q}$ value. Even when initialized with a severely inaccurate value (50 times larger than the truth), the algorithm successfully converges to an accurate $\bar{Q}$ estimate within a short period, and the final RMSE remains close to the optimal case. This demonstrates the effectiveness of the online ML estimation mechanism and reduces the burden of precise initial parameter tuning.

The measurement noise covariance $\bar{R}$ is typically determined by sensor specifications but can be misspecified. We evaluated the performance by scaling the nominal $\bar{R}$ by factors of {0.5,1,2}. The results, summarized in Figure 12c, show that overestimating $\bar{R}$ (using $2\times \bar{R}$) leads to a slightly more conservative filter, with a negligible increase in RMSE. Underestimating $\bar{R}$ (using $0.5\times \bar{R}$) makes the filter overly confident in the measurements, causing a slightly larger performance degradation during GNSS availability periods, but the filter remains stable. Overall, the ICKF demonstrates satisfactory robustness to moderate errors in the $\bar{R}$ parameter.

In conclusion, the sensitivity analysis confirms that the ICKF algorithm is not overly sensitive to its key parameters. The adaptive ML estimation of $\bar{Q}$ effectively mitigates errors in the initial setting. The recommended values are $\bar{N}=10$ and an initial $\bar{Q}$ based on the best available knowledge (e.g., sensor specifications), which can be generously scaled without significant performance loss. The $\bar{R}$ parameter should be set as accurately as possible, but the algorithm can tolerate moderate inaccuracies.

### 4.3. Experiments and Analysis

**Figure 13:**
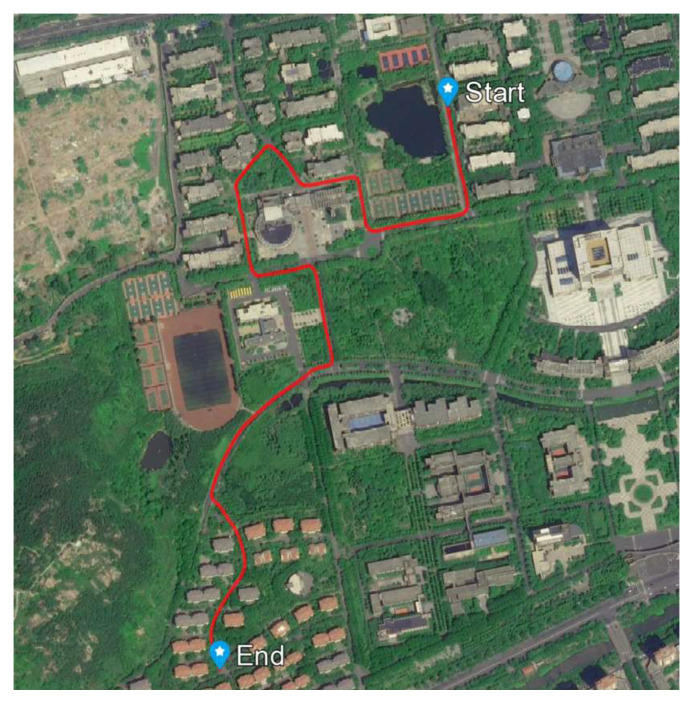
The test trajectory of the vehicle.

To evaluate the performance of the proposed algorithm in real-world complex environments, a car-mounted field test was conducted. The test was carried out on the campus of Shandong University of Science and Technology in Qingdao, Shandong Province, China. The test route is approximately 4 km long. This environment constitutes a typical “urban canyon” where the trajectory is surrounded by buildings and dense trees, leading to frequent GNSS signal interruptions and degradation. The longest continuous GNSS outage during the test lasted approximately 30 s. This provided an ideal scenario to validate the algorithm’s robustness under GNSS-denied conditions. The NovAtel SPAN system was employed to provide a reference trajectory with centimeter-level accuracy, which served as the ground truth. The results of our self-developed algorithm were compared against this reference for objective accuracy assessment. During the test, the vehicle experienced multiple episodes of complete GNSS outages caused by obstructions, effectively simulating the challenges commonly encountered in urban driving. Additionally, the navigation system model utilized is a theoretical approximation of the real dynamic system model, which inherently results in process model uncertainty. The devices for the experiment include the inertial measurement unit (IMU), GNSS, and NovAtel SPAN system. The gyroscope of the IMU has a bias of $1^{\circ }/h$, an angular random walk of $0.071^{\circ }/\surd h$, and a scale factor of 100 ppm, whereas the accelerometer has a bias of 1 mg and a scale factor of 300 ppm. The IMU and GNSS systems operate with sampling frequencies of 200 Hz and 5 Hz, respectively. The NovAtel SPAN system provides a reference trajectory with centimeter-level accuracy, as shown in Figure 13. We set the initial covariance ${\bar{P}}_{0\left|0\right.}$, the initial process noise covariance ${\bar{Q}}_{0\left|0\right.}$, and the covariance of the measurement noise ${\bar{R}}_{k}$ as ${\bar{P}}_{0\left|0\right.}=diag\left(\left[\begin{array}{lllllll} \sigma_{a}^{2}I_{1\times 3} & \sigma_{b}^{2}I_{1\times 3} & \sigma_{c}^{2}I_{1\times 3} & \sigma_{e}^{2}I_{1\times 3} & \sigma_{f}^{2}I_{1\times 3} & \sigma_{g}^{2} & \sigma_{h}^{2} \end{array}\right]\right)$; ${\bar{Q}}_{0\left|0\right.}=diag\left(\left[\begin{array}{lllllll} q_{a}^{2}I_{1\times 3} & q_{b}^{2}I_{1\times 3} & 0_{1\times 3} & q_{e}^{2}I_{1\times 3} & q_{f}^{2}I_{1\times 3} & q_{g}^{2} & q_{h}^{2} \end{array}\right]\right)$; ${\bar{R}}_{k}=\left[\begin{array}{cc} {\left(2.5\;m\right)}^{2}I_{ℑ} & 0_{ℑ\times 2ℑ}\\ 0_{ℑ\times 2ℑ} & {\left(0.1\;\;m/s\right)}^{2}I_{ℑ} \end{array}\right]$. $\sigma_{a}=0.175\;\mathrm{rad}$, $\sigma_{b}=0.1\;m/s$, $\sigma_{c}=10\;m$, $\sigma_{e}=1\;\mathrm{mg}$, $\sigma_{f}=4.85\times 10^{-5}\;\mathrm{rad}/s$, $\sigma_{g}=10\;m$, $\sigma_{h}=0.1\;m/s$, $q_{a}=5.82\times 10^{-6}\;\mathrm{rad}/\surd s$, $q_{b}=0.2\;\mathrm{mg}$, $q_{e}=3.16\times 10^{-4}\;m/s^{2}/\surd s$, $q_{f}=1.41\times 10^{-6}\;\mathrm{rad}/s/\surd s$, $q_{g}=1\;m/\surd s$, $q_{h}=1\;m/s/\surd s$, ℑ represent the number of visible satellites.

**Figure 14:**
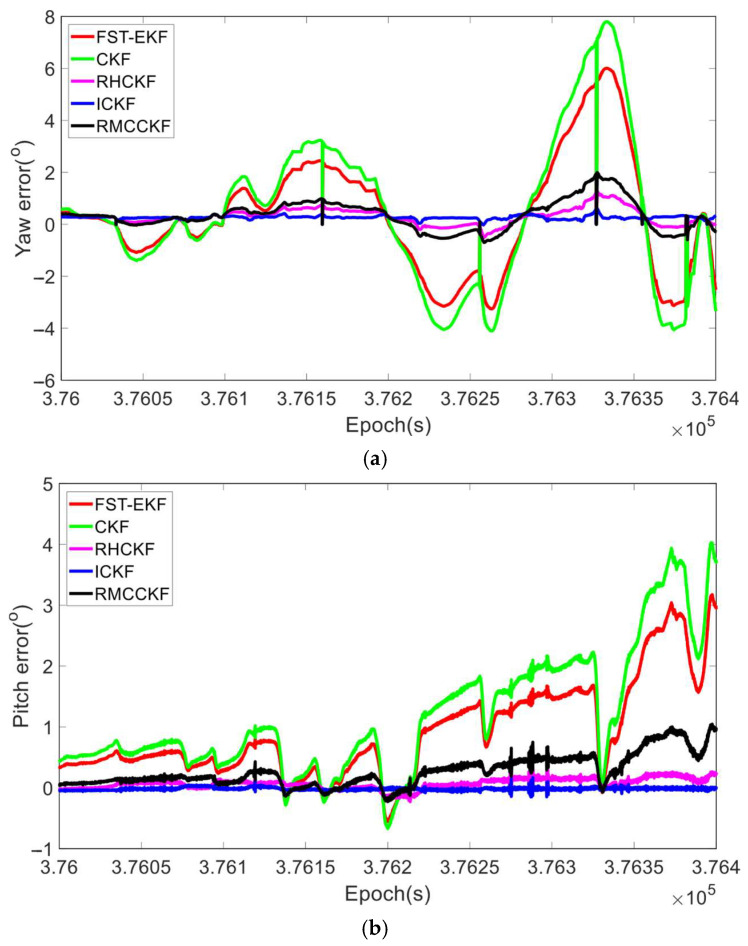
Attitude error of different algorithms. (**a**) Yaw error; (**b**) pitch error.

**Figure 15:**
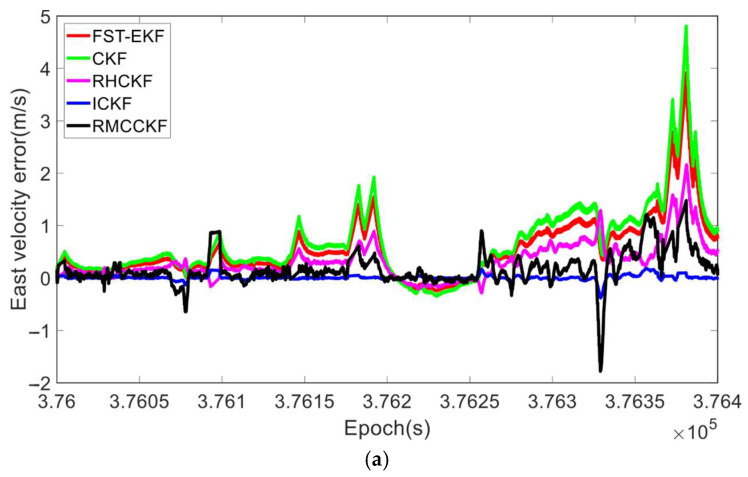

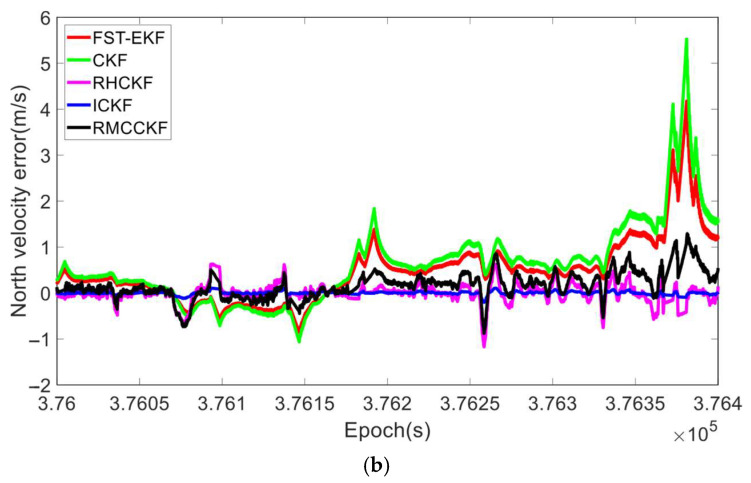
Velocity error of different algorithms. (**a**) East velocity error; (**b**) north velocity error.

**Figure 16:**
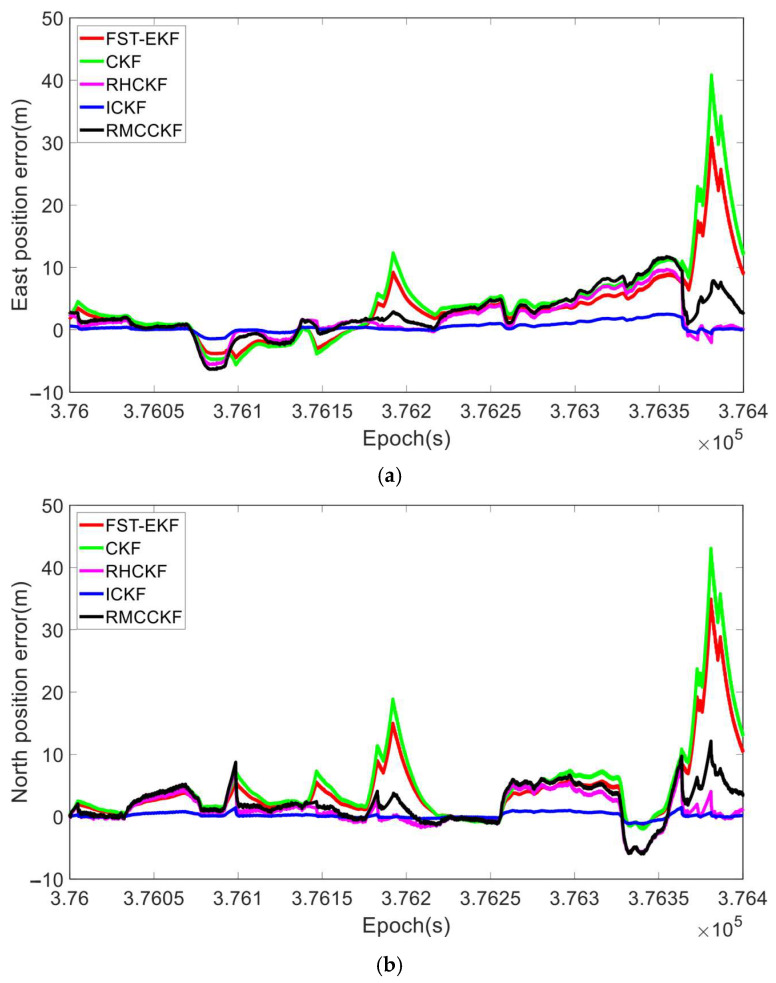
Position error of different algorithms. (**a**) East position error; (**b**) north position error.

Figure 14, Figure 15 and Figure 16 illustrate the errors in attitude, velocity, and position for various algorithms, where the horizontal axis represents the GNSS epoch. Table 6 shows RMSEs of attitude, velocity, and position in the vehicle navigation experiment process. Based on the above experimental results, we can see that the CKF demonstrates relatively poor performance among all categories of filters, exhibiting large RMSE values in yaw angle (2.78°), northward velocity (1.09 m/s), and northward position (8.62 m). The CKF uses a cubature rule to approximate the nonlinear system. However, due to the presence of missing GNSS observations, and system noise uncertainty, the Gaussian assumption of the posterior PDF no longer holds. CKF assumes a Gaussian noise model and uses a fixed process noise covariance, which limits its ability to handle missing observations and model uncertainties. This causes the CKF to diverge during GNSS-denied periods, leading to the high RMSE values in the results. The superior performance of FST-CKF over CKF is mainly due to the introduction of fuzzy logic and a strong tracking mechanism, which can dynamically adjust the filtering gain based on changes in the system state. When GNSS signals are lost or there is process uncertainty, the strong tracking mechanism will accelerate the convergence of the gain to enhance the filter’s response to abrupt changes, while fuzzy logic helps adaptively adjust the filtering parameters, effectively reducing the impact of model mismatch and enhancing the stability and adaptability of the system. However, the FST-EKF also exhibits certain divergence phenomena and performs worse than the RHCKF, ICKF, and RMCCKF, mainly because the FST-EKF relies on measurement updates to correct the system state estimation. When GNSS signals are frequently lost, the filter can only rely on the system’s prediction model for state updates, and the FST-EKF has higher requirements for the accuracy of the system model. If the process model is inaccurate or has strong nonlinearity, the prediction error will accumulate rapidly during the period of GNSS signal loss. In addition, although the strong tracking mechanism can quickly adjust the gain when the signal is recovered, the long-term prediction error caused by frequent GNSS loss will exceed the compensation capacity of the mechanism, ultimately leading to a decline in the filter performance. RHCKF performs significantly better than CKF and FST-EKF, particularly in GNSS-denied environments. It achieves much lower RMSE values, such as 0.43° in yaw angle and 3.48 m in east position errors. RHCKF improves on CKF by using a resampling-free sigma-point update framework, which reduces the impact of missing GNSS observations. This makes it more robust in GNSS-denied scenarios, but it still suffers from a fixed process noise covariance assumption. Without dynamic adaptation, RHCKF’s performance degrades when faced with process model uncertainty, although it handles missing observations better than CKF. RHCKF and RMCCKF leverage robust filtering techniques that reduce the impact of missing observations without the need for resampling. RMCCKF’s use of the maximum correntropy criterion provides additional resilience against outliers by focusing on the most likely measurements, reducing the influence of extreme errors on the state estimation. These features make RMCCKF particularly suited for environments with frequent GNSS disruptions and inconsistent observation quality. Thus, theoretically, RMCCKF’s performance should be superior to RHCKF. However, the experimental results show that in terms of attitude, north velocity, and position, RMCCKF’s performance is slightly inferior to RHCKF. We attribute this to the fact that the kernel bandwidth of the RMCCKF method greatly influences its performance, and since the kernel bandwidth is manually selected through trial and error, it is challenging to achieve an optimal setting. ICKF outperforms all other filters in every category (attitude, velocity, and position). It exhibits significantly lower RMSE values, especially during GNSS outages, and maintains high accuracy even in challenging scenarios with process model uncertainties. The superior performance of ICKF is largely due to its use of the ML principle and the MUF. These techniques enable ICKF to iteratively update the process noise covariance in real time, making it highly adaptive to both system noise uncertainty and missing GNSS observations. The ML principle allows ICKF to better handle nonlinear dynamics, providing more accurate estimates of yaw, velocity, and position, even during GNSS-denied periods. Additionally, the posterior cubature point error matrix in ICKF improves the filter’s robustness by reducing the sensitivity to the errors accumulated during long GNSS outages.

## 5. Conclusions

This paper presents a novel approach to enhance the performance of the CKF, known as the improved cubature Kalman filter (ICKF). By implementing a modified cubature point update framework that minimizes the impact of missing observations, the ICKF significantly improves the reliability of the filtering process. Additionally, the ICKF utilizes the maximum likelihood principle to accurately estimate and update the process noise covariance. The study involved simulation experiments and car-mounted INS/GNSS navigation field tests to compare the performance of the ICKF with other Kalman filters, including the FST-EKF, CKF, RHCKF, and RMCCKF, across various scenarios. The results indicate that, in the presence of missing GNSS observations and system noise uncertainty, the ICKF achieves superior filtering accuracy and stability.

Of course, the method we propose also has certain limitations. For example, (1) the ICKF involves complex noise covariance adaptation, which results in high computational complexity and processing time, and may not be suitable for systems with high real-time requirements; (2) the experimental results of this study have verified the effectiveness of the ICKF in specific data sets and experimental conditions, but its effectiveness in more widespread applications, such as different types of platforms, other sensor combinations, etc., still needs to be further verified and optimized. Therefore, our future research will focus on enhancing the proposed ICKF in the following areas: (1) integrating artificial intelligence techniques to reduce the algorithm’s computational complexity and to adaptively determine the size of the fixed-length memory estimation window in different application scenarios; (2) conducting a qualitative analysis of the improvement in process noise covariance estimation with higher precision INS parameters.

## Figures and Tables

**Table 1:** Simulation parameters.

Parameter	Value	Parameter	Value
Constant drift of gyro	$10^{\circ }/h$	Noise of gyro	$0.1^{\circ }/\surd h$
Bias of accelerometer	1 mg	Noise of accelerometer	0.1 mg/√Hz
Equivalent pseudo-range error of GNSS	10 km	Equivalent pseudo-range rate error of GNSS	100 m/s
Initial attitude error	$\left(-0.05^{\circ },0.04^{\circ },5^{\circ }\right)$	Update rate of GNSS	2 Hz
Update rate of INS	100 Hz	Type of aircraft	fighter plane
The geometric characteristics of the aircraft	9.96 m (wing span), 3.56 (aspect ratio), 27.87 $m^{2}$ (wing area), 2.8 m (mean chord), $35^{\circ }$ (sweep angle), 15.06 m (Length)	The weight characteristics of the aircraft	19,200 kg (max takeoff weight), 9500 kg (empty weight), 5000 kg (max fuel weight), 1.05 (thrust-to-weight)
The aerodynamic characteristics of the aircraft	1.8 (maximum lift coefficient), 0.02 (zero-lift drag coefficient), 0.04 (drag coefficient varies with lift coefficient), 2 (maximum Mach number), 670 m/s (maximum flight speed)	The stability derivatives of the aircraft	−0.3 (roll damping derivative), 0.2 (roll rate derivative), −0.5 (pitch damping derivative), −0.8 (angle of attack derivative), −0.4 (yaw damping derivative), 0.1 (yaw coupling derivative)

**Table 2:** The ${\mathrm{RMSE}}_{p}$ of the position of different algorithms throughout the simulation.

Algorithm	Environment 1 (m)	Environment 2 (m)	Environment 3 (m)
FST-EKF	96.03	377.63	1001.59
CKF	119.53	457.93	1198.53
RHCKF	59.10	221.80	620.86
ICKF	12.21	54.20	169.44
RMCCKF	24.14	92.26	286.26

**Table 3:** The ${\mathrm{RMSE}}_{p}$ of the position of different algorithms in the simultaneous presence of process model uncertainty and GNSS-denied environment.

Algorithm	Environment 1 (m)	Environment 2 (m)	Environment 3 (m)
FST-EKF	245.73	957.56	1256.16
CKF	306.89	1211.53	1665.34
RHCKF	169.88	436.54	1108.95
ICKF	30.45	87.32	268.72
RMCCKF	45.98	195.81	477.26

**Table 4:** Consistency index analysis based on state error covariance (during GNSS outage).

Algorithm	East Position (%)	North Position (%)	Yaw Angle (%)	Average (%)
FST-EKF	78.3	76.9	79.1	78.1
CKF	72.6	70.8	74.3	72.6
RHCKF	85.2	83.7	86.1	85.0
ICKF	96.8	95.2	97.1	96.4
RMCCKF	89.7	88.4	90.2	89.4

**Table 5:** The ${\mathrm{RMSE}}_{p}$ of the position of different algorithms.

Algorithm	Environment 1 (m)	Environment 2 (m)	Environment 3 (m)
FST-EKF	266.54	981.12	1380.31
CKF	300.21	1221.45	1718.96
RHCKF	156.32	412.84	1055.45
ICKF	68.69	182.31	599.61
RMCCKF	103.71	288.73	865.56

**Table 6:** RMSEs of different algorithms.

Algorithm	Attitude (°)		Velocity (m/s)		Position (m)	
	Yaw Angle	Pitch Angle	East	North	East	North
FST-EKF	2.20	1.13	0.79	0.85	6.40	6.83
CKF	2.78	1.48	0.98	1.09	8.39	8.62
RHCKF	0.43	0.08	0.47	0.21	3.48	2.66
ICKF	0.27	0.03	0.06	0.04	0.91	0.51
RMCCKF	0.66	0.35	0.36	0.33	4.04	3.56

## Data Availability

The original contributions presented in this study are included in the article. Further inquiries can be directed to the corresponding author(s).

## References

[1] Xu Y., Wan D., Shmaliy Y.S., Chen X.Y., Shen T., Bi S.H. (2024). Dual free-size LS-SVM assisted maximum correntropy Kalman filtering for seamless INS-based integrated drone localization. IEEE Trans. Ind. Electron..

[2] Huang H.Q., Wei J.Y., Wang D., Zhang L., Wang B. (2022). In-motion initial alignment method based on vector observation and truncated vectorized K-matrix for SINS. IEEE Trans. Instrum. Meas..

[3] Qin H.M., Wang Y., Wang G.C., Qin X.H., Bian Y.G. (2023). GSCV-XGBoost based information reconstruction and fusion method for SINS/DVL integrated navigation system. Meas. Sci. Technol..

[4] Qu C.Y., Li J.L., Zhang W. (2022). Improved integrated navigation method of micro position and orientation system based on installation error angle calibration. Meas. Sci. Technol..

[5] Liu Y.H., Ning X.L., Li J.L., Ye W., Wang B., Ma X. (2021). Adaptive central difference Kalman filter with unknown measurement noise covariance and its application to airborne POS. IEEE Sens. J..

[6] Li J.L., Zou S.Y., Li Y.Q. (2019). A nonlinear two-filter smoothing estimation method based on DD2 filter for land vehicle POS. Meas. Sci. Technol..

[7] Liu D., Chen X.Y., Xu Y., Liu X., Shi C.F. (2019). Maximum correntropy generalized high-degree cubature Kalman filter with application to the attitude determination system of missile. Aerosp. Sci. Technol..

[8] Ning X.L., Gui M.Z., Fang J.C., Liu G. (2017). Differential X-ray pulsar aided celestial navigation for Mars exploration. Aerosp. Sci. Technol..

[9] Wang D., Wang B., Huang H.Q., Zhang H.X. (2024). A SINS/DVL navigation method based on hierarchical water velocity estimation. Meas. Sci. Technol..

[10] Huang H.Q., Tang J.C., Liu C., Zhang B., Wang B. (2021). Variational Bayesian-based filter for inaccurate input in underwater navigation. IEEE Trans. Veh. Technol..

[11] Gao B.B., Hu G.G., Zhong Y.M., Zhu X.H. (2022). Distributed state fusion using sparse-grid quadrature filter with application to INS/CNS/GNSS integration. IEEE Sens. J..

[12] Song L.H., Song L.J. (2025). Estimation of navigation receiver clock bias utilizing wavelet transform and long short-term memory network models: A clock-aided positioning algorithm with validation of results using GPS data. Measurement.

[13] Guo X.T., Shen C., Tang J., Li J., Liu J. (2021). A fusion strategy for reliable attitude measurement using MEMS gyroscope and camera during discontinuous vision observations. Mech. Syst. Signal Process..

[14] Xu Y., Yu J.W., Wang X.P., Li T., Sun M.X. (2024). Dual foot-mounted localisation scheme employing a minimum-distance-constraint Kalman filter under coloured measurement noise. Micromachines.

[15] Liu F.Y., Sun X.H., Xiong Y.F., Huang H.Q., Guo X.T., Zhang Y., Shen C. (2019). Combination of iterated cubature Kalman filter and neural networks for GPS/INS during GPS outages. Rev. Sci. Instrum..

[16] Shen C., Xiong Y.F., Zhao D.H., Wang C.G., Cao H.L., Song X., Tang J., Liu J. (2022). Multi-rate strong tracking square-root cubature Kalman filter for MEMS-INS/GPS/polarization compass integrated navigation system. Mech. Syst. Signal Process..

[17] Gao B.B., Hu G.G., Zhong Y.M., Zhu X.H. (2021). Cubature rule-based distributed optimal fusion with identification and prediction of kinematic model error for integrated UAV navigation. Aerosp. Sci. Technol..

[18] Wang G.C., Xu X.S., Zhang T. (2020). M-M estimation-based robust cubature Kalman filter for INS/GPS integrated navigation system. IEEE Trans. Instrum. Meas..

[19] Hu G.G., Gao B.B., Zhong Y.M., Gu C.F. (2020). Unscented kalman filter with process noise covariance estimation for vehicular ins/gps integration system. Inf. Fusion.

[20] Meng Y., Gao S.S., Zhong Y.M., Hu G.G., Subicc A. (2016). Covariance matching based adaptive unscented Kalman filter for direct filtering in INS/GNSS integration. Acta Astronaut..

[21] Xiong Y.F., Zhang Y., Guo X.T., Wang C.G., Shen C., Li J., Tang J., Liu J. (2019). Seamless global positioning system/inertial navigation system navigation method based on square-root cubature Kalman filter and random forest regression. Rev. Sci. Instrum..

[22] Zhang Y.X. (2019). A fusion methodology to bridge GPS outages for INS/GPS integrated navigation system. IEEE access.

[23] Wu Q.D., Li C.X., Shen T., Xu Y. (2023). Improved adaptive iterated extended Kalman filter for GNSS/INS/UWB-integrated fixed-point positioning. CMES-Comput. Model. Eng. Sci..

[24] Zhang Y.X., Wang L.H. (2019). A hybrid intelligent algorithm DGP-MLP for GNSS/INS integration during GNSS outages. J. Navig..

[25] Ye W., Li J.L., Fang J.C., Yuan X.Z. (2018). EGP-CDKF for performance improvement of the SINS/GNSS integrated system. IEEE Trans. Ind. Electron..

[26] Chen W., Li X., Song X., Li B., Song X.H., Xu Q.M. (2015). A novel fusion methodology to bridge GPS outages for land vehicle positioning. Meas. Sci. Technol..

[27] Li X., Xu Q.M. (2017). A reliable fusion positioning strategy for land vehicles in GPS-denied environments based on low-cost sensors. IEEE Trans. Ind. Electron..

[28] Chen L.Z.T., Fang J.C. (2014). A hybrid prediction method for bridging GPS outages in high-precision POS application. IEEE Trans. Instrum. Meas..

[29] Shen C., Zhang Y., Tang J., Cao H.L., Liu J. (2019). Dual-optimization for a MEMS-INS/GPS system during GPS outages based on the cubature Kalman filter and neural networks. Mech. Syst. Signal Process..

[30] Doostdar P., Keighobadi J., Hamed M.A. (2019). INS/GNSS integration using recurrent fuzzy wavelet neural networks. GPS Solutions.

[31] Yao Y.Q., Xu X.S., Zhu C.C., Chan C.Y. (2017). A hybrid fusion algorithm for GPS/INS integration during GPS outages. Measurement.

[32] Wang G.W., Cui B.B., Tang C.Y. (2022). Robust cubature Kalman filter based on maximum correntropy and resampling-free sigma-point update framework. Digital Signal Process..

[33] Wang J., Zhang T., Xu X., Li Y. (2018). A variational Bayesian based strong tracking interpolatory cubature Kalman filter for maneuvering target tracking. IEEE Access.

[34] Chen Z.H., Liu Y.X., Liu S.G., Wang S.L., Yang L. (2025). An improved fading factor-based adaptive robust filtering algorithm for SINS/GNSS integration with dynamic disturbance suppression. Remote Sens..

[35] Sage A.P., Husa G.W. Adaptive Filtering with Unknown Prior Statistics. Proceedings of the 1969 IEEE Conference on Decision and Control.

[36] Sahl S., Song E.B., Niu D.B. (2024). Robust cubature Kalman filter for moving-target tracking with missing measurements. Sensors.

[37] Cui B.B., Wei X.H., Chen X.Y., Wang A.C. (2021). Improved high-degree cubature Kalman filter based on resampling-free sigma-point update framework and its application for inertial navigation system-based integrated navigation. Aerosp. Sci. Technol..

[38] Li L., Xia Y.Q. (2012). Stochastic stability of the unscented Kalman filter with intermittent observations. Automatica.

[39] He K.H., Dong C.Y. A fuzzy strong tracking extended Kalman filter for UAV navigation considering interruption of GPS signal. Proceedings of the IEEE International Conference on Power, Intelligent Computing and Systems.

